# Research on cluster system distribution of traditional fort-type settlements in Shaanxi based on K-means clustering algorithm

**DOI:** 10.1371/journal.pone.0264238

**Published:** 2022-03-11

**Authors:** Xuan Wang, Anyang Shen, Xin Hou, Lifeng Tan

**Affiliations:** 1 School of Architecture, Tianjin University, Tianjin, China; 2 School of Architecture, Tianjin Chengjian University, Tianjin, China; Northeastern University (Shenyang China), CHINA

## Abstract

Taking the traditional fort-type settlements in Shaanxi as the research object, quantitative research methods such as K-means clustering algorithm, correlation analysis, density analysis, and nearest neighbor index are used to study their spatial distribution, formation causes, and cluster characteristics. The objective of the study is to break through the geographical limitations of fort-type settlements research and to explore the scientific methods of classifying and analyzing traditional fort-type settlements. The conclusions are: (1) The results of cluster analysis show that the fort-type settlements in Shaanxi can be divided into three categories; (2) The overall distribution of fort-type settlements in Shaanxi shows multi-point aggregation, and contains both point and linear aggregation distribution; (3) There are four typical cluster systems among the traditional fort-type settlements in Shaanxi; (4) The factors that have the greatest influence on the distribution of settlements are construction force, wall masonry, age, fortification purpose, and topographic environment. The article innovatively proposes the "cluster system" perspective and introduces mathematical algorithms and quantitative research methods to study the cluster system of the fort-type Settlements. This approach is feasible and can be applied to other settlement-related studies. At the same time, the perspective of cluster system could be used in heritage conservation, which can contribute to the restoration of architectural relics and systemic conservation on a larger scale.

## Introduction

Fort-type settlements are typical defensive settlements widely existing in China, whose construction originates from human instability in the environment and is mainly characterized by linear fortification of the periphery [1]. A large number of folk immovable artifacts were discovered in China’s third cultural relics census (2007–2011) and academic field surveys over the past several decades, including many fort-type settlements. The value of the cluster system is affirmed in *The Charter on the Built Vernacular Heritage*. “The vernacular is only seldom represented by single structures, and it is best conserved by maintaining and preserving groups and settlements of a representative character, region by region” [2]. In this context, this study proposes to study and protect the fort-type settlements from the perspective of "group system" and explore a more systematic approach to heritage conservation.

Since the beginning of the twenty-first century, academics began to gradually divide fort-type settlements from the overall settlements studies to form an independent research field, and the relevant results covered various provincial and municipal areas such as Shanxi, Henan, and Hubei. After more than ten years of development, several sub-topic research directions have been deepened and formed, including the study of military fort-type settlements with the Great Wall as a link [3] and the study of fort-type settlements system with the marine defense as a clue [4, 5], etc. In other disciplines such as history and archaeology, the study of fort-type settlements is mainly based on historical background, and the results include historical changes and social structure of fort-type settlements [6] and excavation of fort-type sites [7]. With the arrival of the bottleneck period, the whole field of settlements research has taken a step ahead in the macro direction, and scholars have gradually tried to analyze architectural settlements from the perspective of group genealogy in recent years, including zoning from the perspective of traditional architecture [8], and introducing genealogical research from other disciplinary approaches such as landscape gene theory [9] and language theory [10]. In the wider field of architecture and urban planning, scholars have started to try to apply mathematical algorithms to architectural research by introducing models such as RBF neural network model [11] and fuzzy cellular automata [12]. However, similar studies have not been widely carried out in the study of fort-type settlements. In general, the research for fort-type settlements remains in the traditional field of historical development and architectural paradigm, while group system research is confined based on subjective experience, lacking strong data support and objective analysis tools.

Therefore, to avoid the problem that research of cluster systems is too subjective, to provide a practical quantitative method for the classification of cluster systems, and to deeply explore the main reasons and typical characteristics of cluster system formation, this paper introduces quantitative analysis methods such as cluster analysis, correlation analysis, principal component analysis, kernel density estimation, and nearest neighbor index, relying on ArcGIS and Python platform, to study the fort-type settlements in Shaanxi. The innovation of this study lies in the introduction of the "cluster system" perspective and the application of mathematical algorithms in the study of cluster systems. Finally, this paper divides the fort-type settlements in Shaanxi into four clusters with different formation contexts and distinctive characteristics, proposes the heritage conservation of fort-type settlements as cluster systems, and provides a theoretical basis for the restoration of architecture.

## Materials and methods

### Research object and data sources

**Fig 1:**
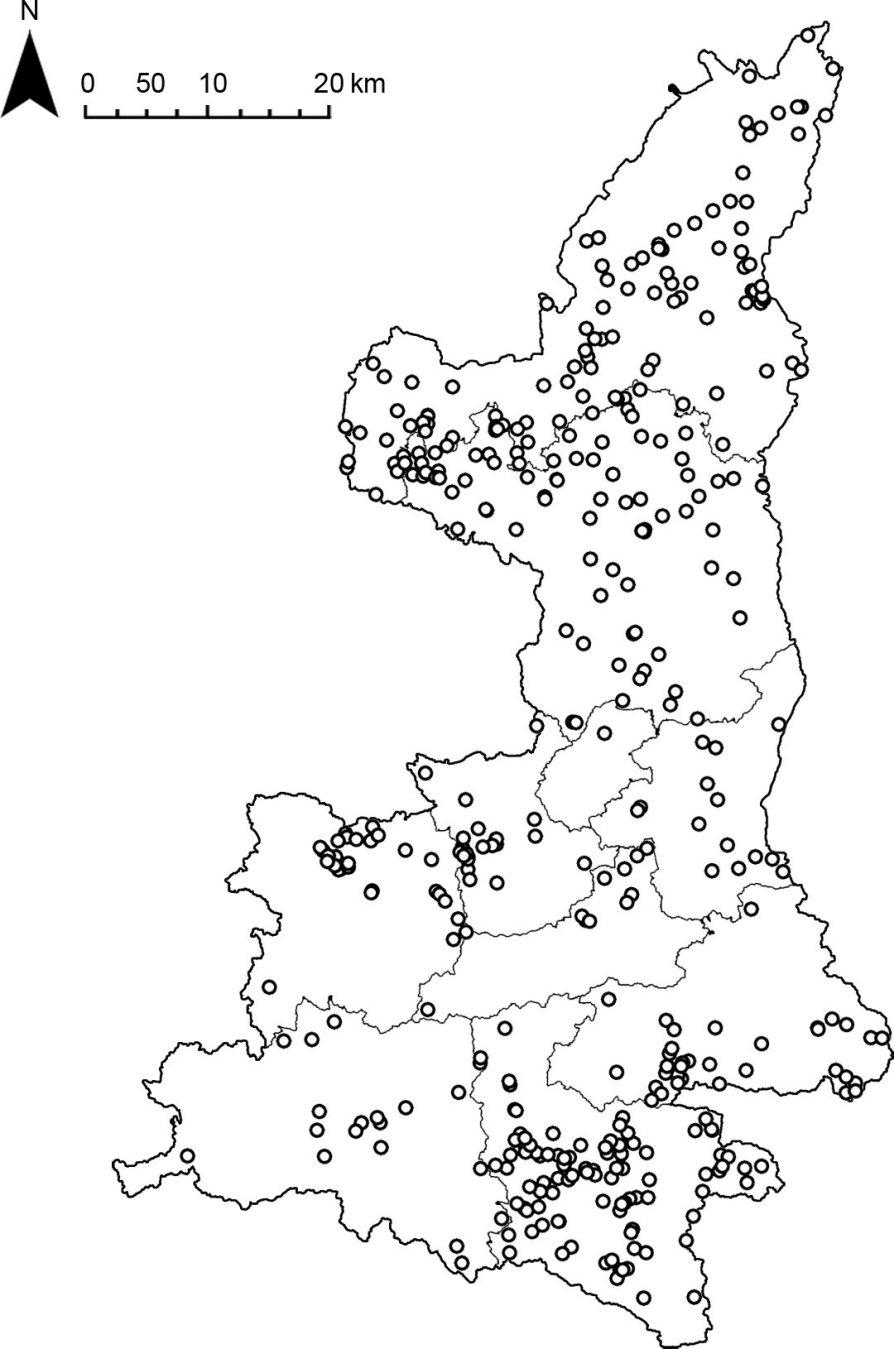
Distribution map of fort-type settlements in Shaanxi.

The study area was defined as Shaanxi Province, which was one of the most severely warring regions in China since ancient times, with many fort-type settlements and complex composition (Fig 1). The administrative division data of Shaanxi province were obtained from the National Geographic Information Resources Catalogue Service System, and the main fort-type settlements data were obtained from *The National Bureau of Cultural Relics Atlas of Chinese Cultural Relics Shaanxi Sub-volume* [13], which were surveyed from 1956 to 1981. The supplementary data of the fort-type settlements were from the third cultural relics census and fieldwork (2007–2021). On this basis, data cleaning was carried out to remove data with unknown impact factor attributes, and finally a total of 409 fort-type settlements data. ArcGIS10.2 was used for data import and cleaning, etc., and Python was used for data analysis and calculation work, and all data were in coordinate system GCS_Beijing_1954.

### Research methods

**Fig 2:**
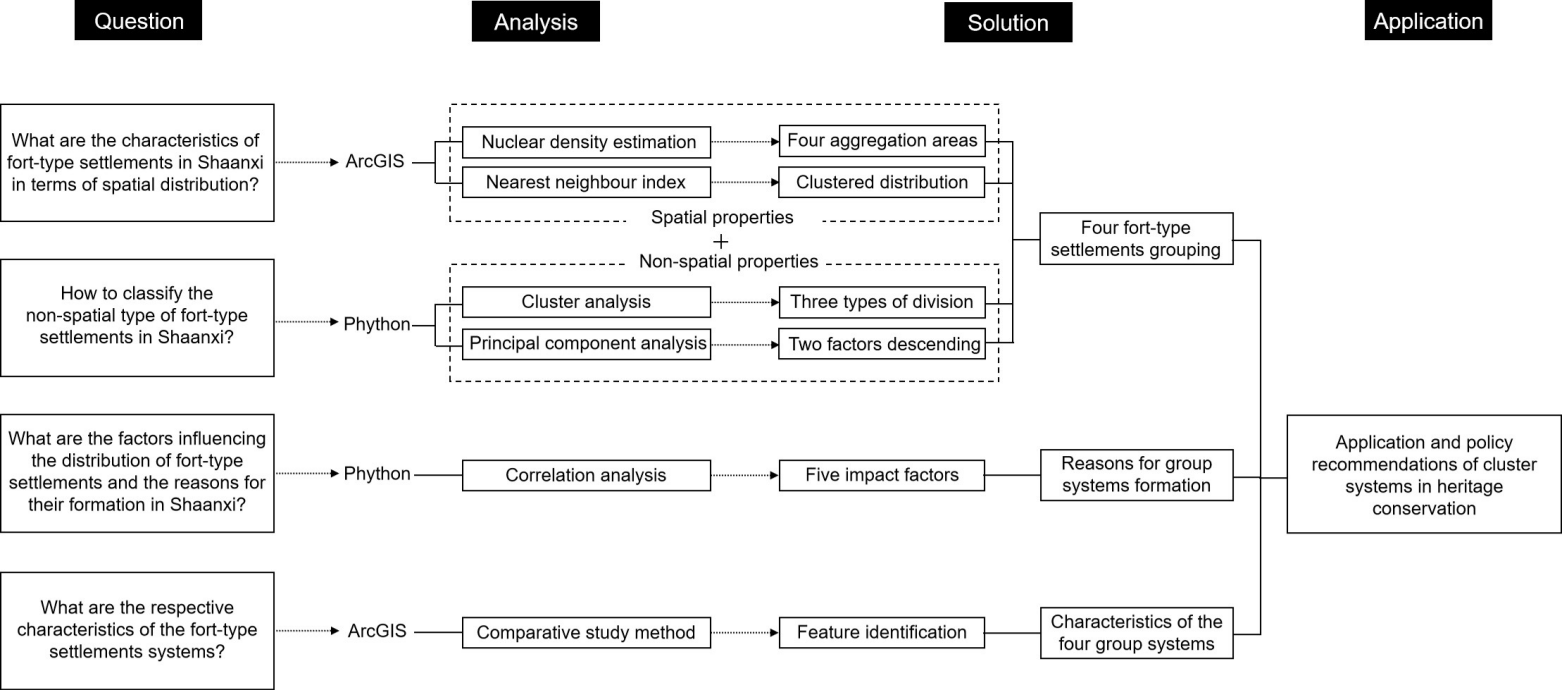
Research framework diagram.

The research ideas are as follows (Fig 2): Firstly, the nearest neighbor index and density analysis methods are used to study the spatial distribution characteristics of the fort-type settlements in Shaanxi, and to discover where the fort-type settlements are most densely distributed. Meanwhile, the K-means clustering algorithm and principal component analysis are used to study the non-spatial attributes of the clusters and classify them into different types. Then the type points are superimposed with the aggregated distribution areas, and the resulting cluster of fort-type settlements has both geographic aggregation and typological similarity, which is called the fort-type cluster system. Secondly, the correlation analysis method is used to study the factors influencing the distribution and causes of the formation of the fort-type cluster, and five main influencing factors are found. Thirdly, the typical characteristics of each fort-type cluster are analyzed by the comparative study method. Finally, it is found that each fort-type cluster can be used as a theoretical basis for heritage conservation and applied to the restoration of cultural relics.

#### Cluster analysis method

Cluster analysis is the process of dividing a collection of data objects into clusters such that the objects within the clusters are similar to each other and the objects between the clusters are not similar, and its goal is to discover the natural groupings of data collections [14]. In this paper, the K-means algorithm is used to calculate the digitally coded fort-type clusters in the Shaanxi region and classify them according to the degree of similarity of indicators between clusters. The formula (1) is as follows.

$$SSE(C)=\sum_{k=1}^{k}{\sum_{x_{i}\subseteq c_{k}}\left\|x_{i}-c_{k}\right\|}^{2}$$ (1)

SSE is the algorithm objective function and c_k_ is the center point of cluster C_k_.

#### Principal component analysis method

The Principal component analysis is a multivariate statistical method that transforms a number of indicators into a few composite indicators by means of dimensionality reduction, in order to facilitate the subsequent analysis to reveal the patterns among internal variables more easily. It is assumed that X_1_, X_2_, …, X_p_ denote the p indicators involved in the study of things, Y_1_, Y_2_, …, Y_p_ represent the p principal components of the original variables after linear transformation, respectively, the mean of the random vector X is μ and the covariance matrix is ∑ [15]. The formula (2) is as follows.


$$\left\{\begin{array}{c} Y_{1}=\mu_{11}X_{1}+\mu_{21}X_{2}+\ldots +\mu_{p1}X_{p}\\ Y_{2}=\mu_{21}X_{1}+\mu_{22}X_{2}+\ldots +\mu_{p2}X_{p}\\ \ldots \\ Y_{p}=\mu_{1p}X_{1}+\mu_{2p}X_{2}+\ldots +\mu_{pp}X_{p} \end{array}\right.$$ (2)

#### Correlation analysis method

Correlation analysis is a multivariate analysis method that studies the overall linear correlation between variables and measures the degree of correlation of variables with the help of correlation coefficients. Let x = (X_1_ X_2_, … X_p_)’ and y = (Y_1_ Y_2_, … Y_p_)’ be two interrelated random variables in which a number of composite variables U_i_, V_i_, respectively, are selected so that each composite variable is a linear combination of the original variables [15]. The formula (3) is as follows.


Ui=ai1X1+ai2X2+…+aipXp=a'xVi=bi1Y1+bi2Y2+…+bipYp=b'yρ(a1'x,b1'y)=maxρ(a'x,b'y)var(a'x)=var(b'y)=1 (3)

#### Kernel density estimation method

The kernel density estimation method is based on the principle that geographic events have a high probability of occurrence in regions with high spatial point density and a low probability of occurrence in regions with low density [16]. The kernel density analysis can calculate the density of fort-type settlements in different areas of Shaanxi Province, and thus discover the area where the cluster system is located. The formula (4) is as follows.

$${\text{f}}_{\text{n}}\left(\text{x}\right)=\frac{1}{\text{nh}}\sum_{\text{i}=1}^{\text{n}}\left[\text{K}\left(\frac{\text{d}\left(\text{x},{\text{x}}_{\text{i}}\right)}{\text{h}}\right)\right]$$ (4)

n denotes the number of spatial entities contained in the distance threshold range, K () denotes the kernel density equation, h denotes the distance threshold, and d(x, xi) denotes the Euclidean distance between two points.

#### Nearest neighbor point index

The nearest point index indicates the mutual proximity of points in space [17] and can be generally classified into three modes: random distribution, cluster distribution, and disperse distribution [18]. The formula (5) is as follows.

$$\text{R}=\frac{\bar{{\text{r}}_{1}}}{\bar{{\text{r}}_{\text{E}}}}=2\sqrt{\text{D}}$$ (5)

$\bar{{\text{r}}_{1}}$ is the actual nearest neighbor distance, $\bar{{\text{r}}_{\text{E}}}$ is the theoretical nearest neighbor distance, and D is the point density.

#### Comparative research method

The comparative research method is the method to discover the similarities and differences between two and more things by comparing them [19]. In this paper, cross-sectional comparison of four fort-type clusters in Shaanxi is conducted separately to summarize their respective typical characteristics.

### Quantitative index system

There are different academic views on the selection of indicator factors for settlements. Wen-qing Wang discussed the zoning of traditional Chinese dwellings from both humanistic and natural aspects [20, 21]. Xiu-ying Shen believed that the formation of settlements was influenced by three factors such as geographical environment, local culture, and architectural materials [22]. Pei-lin Liu believed that settlements genes could be identified by factors such as overall layout characteristics, residential characteristics, cultural symbols, main public buildings, environmental factors, and basic forms [23]. Among these factor divisions, the most frequent ones were geographic environment, colony morphology, spatial structure, architectural layout, and intangible culture [24]. Overall, it is generally accepted to classify influencing factors of settlements into humanistic, natural, and architectural aspects, and the selection of subdivided influencing factors varies with the object of study.

Based on existing studies and the consideration of a comprehensive reflection of the attributes of fort-type settlements, this paper divided the influence factors into three categories: historical and social attributes, physical and geographical attributes, and architectural attributes, reflecting the influence of historical background and geographical environment on the distribution of settlements, and the inherent structure of architecture, respectively (Table 1). In the selection of specific influencing factors, in addition to the basic factors of settlements classification (age, topographic environment, planar form, scale, etc.), defense-related influencing factors (fortification purpose, construction force, defense facilities, etc.) were added in order to reflect the basic characteristics of fort-type settlements in a more comprehensive manner. On this basis, we coded and digitized the data for subsequent computation in Python.

**Table 1:** Quantitative index factors.

Attributes	Impact Factors		Categories
Historical Social Attributes	Age (A1)		Shang、Zhou、Spring and Autumn Period、Warring States、Qin、Han、Northern and Southern Dynasties、Sui、Tang、Five Dynasties、Sixteen Kingdoms Period、Song、Western Xia、Jin、Yuan、Ming、Qing
	Fortification Purpose (A2)		Chengyi、Military Defense、Song-Xia War、Ming-Mongolian War、The White Lotus Uprising、Muslim Uprising
	Construction Force (A3)		Official Construction、Democratic Construction
	Preservation Level (A4)		National Level、District Level、Provincial Level、Municipal Level、County Level、None
Physical Geographic Attributes	Topographic Environment (B1)		Flatland、Mountain、Terrace、Waterfront、Facing the Ditch、the Great Wall
	Planar Form (B2)		Rectangular、Square、Oval、Round、Trapezoid、Triangle、Back、Irregular
Settlement Attributes	Wall Masonry (C1)		Rammed Construction、Stone、Rammed Boulder、Rammed Bricks、Mountains Barrier
	Scale (C2)		101–500、501–1000、1001–5000、5001–10000、10001–100000、100001–1000000、1000001–10000000、10000001–100000000
	Defense Facilities	City Gate (C3)	0–12
		Horse Face (C4)	0–98
		City Trench (C5)	0–1
		Corner House (C6)	0–4
		Beacon (C7)	0–2
		Urn City (C8)	0–4

## Results and discussion

### Division of cluster system in Shaanxi

#### Study on non-spatial properties of fort-type settlements based on cluster analysis and principal component analysis

In order to study the differences in the category characteristics of fort-type settlements in Shaanxi, this paper used the K-Means algorithm to perform cluster analysis. The samples were divided into K clusters, and each cluster was represented by the mean value of all samples in the cluster, which was called the "center of mass". The overall process was as follows [18].

Firstly, K points from the sample were selected as the initial center of mass.

Secondly, the distance of each sample to each center of mass was calculated, and the samples were divided into clusters corresponding to the nearest center of mass.

Thirdly, calculated the mean value of all samples in each cluster, and updated the center of mass of the cluster using this mean value.

Fourthly, repeated steps 2 and 3 until the end of one of the following conditions were reached.

- The change in the position of the center of mass was less than a specified threshold
- The maximum number of iterations was reached

Fifthly, calculated the total distance squared error (SSE) and the percentage of total distance squared error (%SSE).

**Fig 3:**
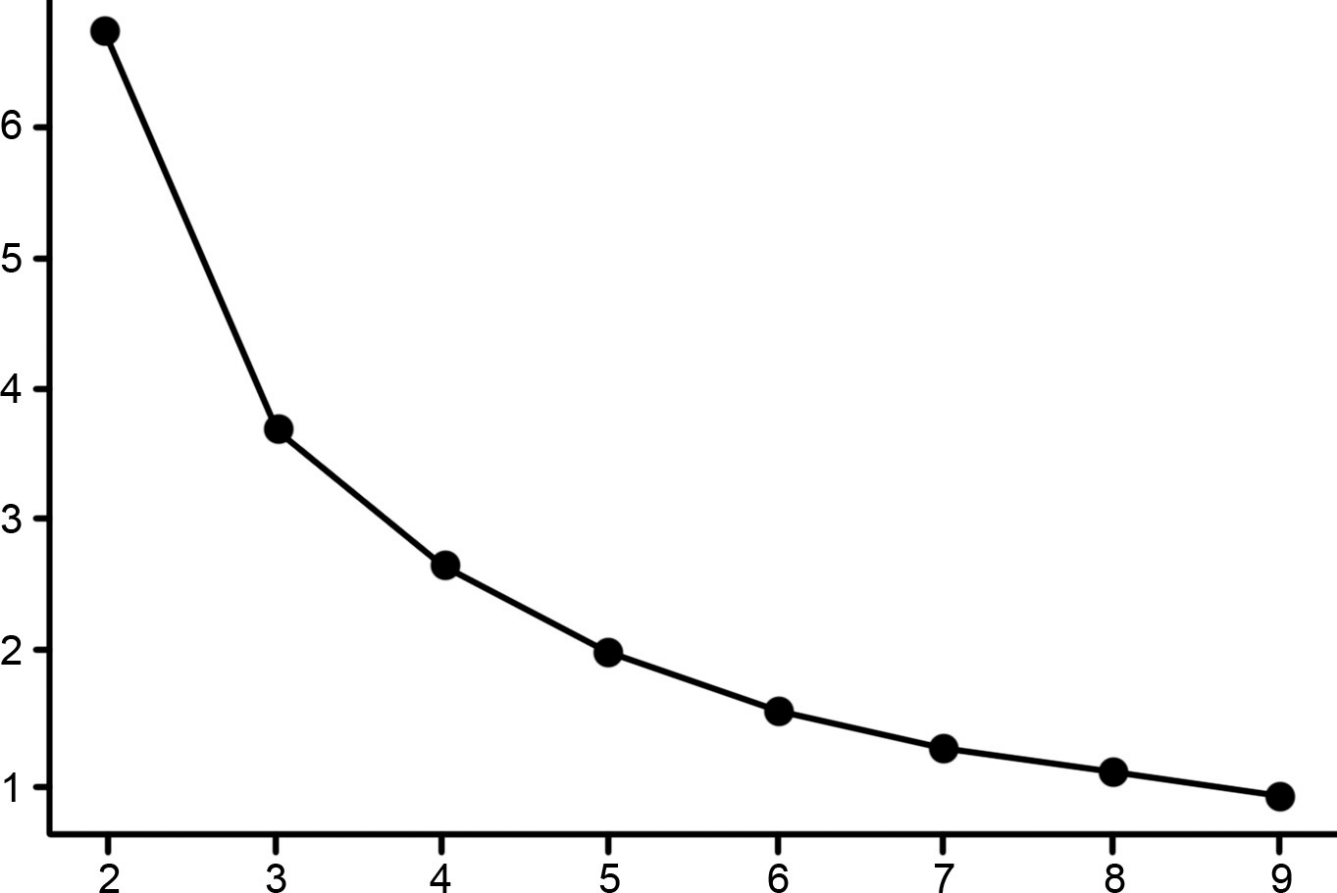
Clustering analysis line chart.

Finally, the optimal number of groupings was determined. The X-axis of the graph (Fig 3) is the number of clustering groups, the Y-axis is the logarithm of the total distance squared error percentage (log%SSE), and the number of groups is determined based on the elbow points of the graph. The results of the clustering analysis show that the best results are obtained when the grouping is three categories.

**Fig 4:**
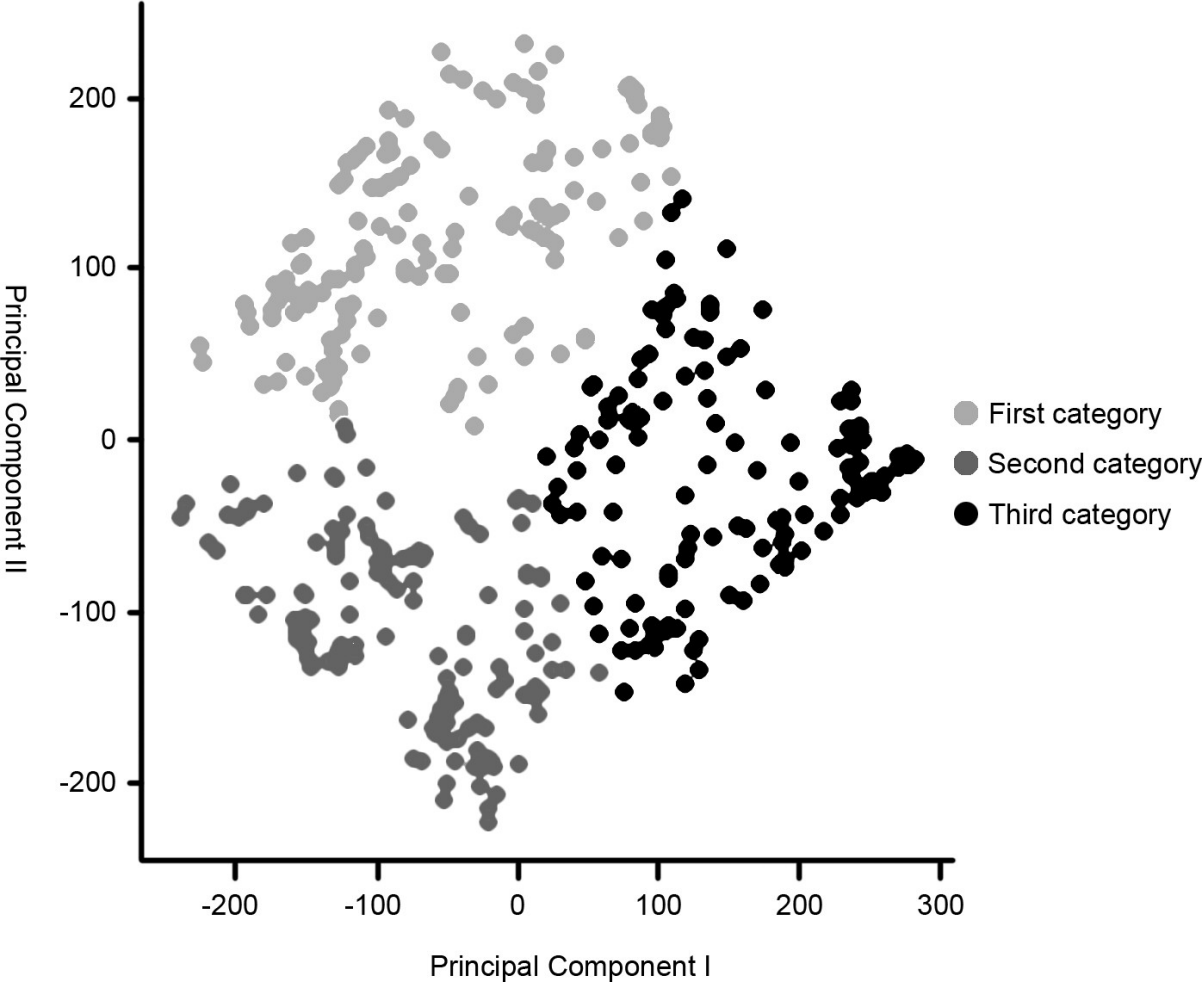
Clustering analysis scatters plot.

The data of the fort-type clusters in the Shaanxi region had a total of 14 influencing factors for three attributes, which were processed by dimensionality reduction analysis using principal component analysis (Table 2) and mapped to a two-dimensional plane in order to plot the individual cluster points into the graph (Fig 4). It can be seen that the three types of forts present obvious clustering effects in the two-dimensional coordinate system, supporting the validity of the clustering results.

**Table 2:** Analysis of the average nearest neighbor of the different cluster systems.

Cluster system	ANN	Z	P	Feature
Qing White Lotus Uprising Democratic Fort Cluster	0.645687	-7.758074	0.000000	Significant aggregation
Qing Muslim Uprising Democratic Earth Fort Cluster	0.661813	-3.602206	0.000316	aggregation
Song-Xia War Border Military Fort Cluster	0.398670	-20.449721	0.000000	Significant aggregation
Ming Great Wall Military Defense System Fort Cluster	0.367674	-34.470703	0.000000	Significant aggregation

#### Study on spatial properties of fort-type settlements based on the nearest neighbor index and density analysis

**Fig 5:**
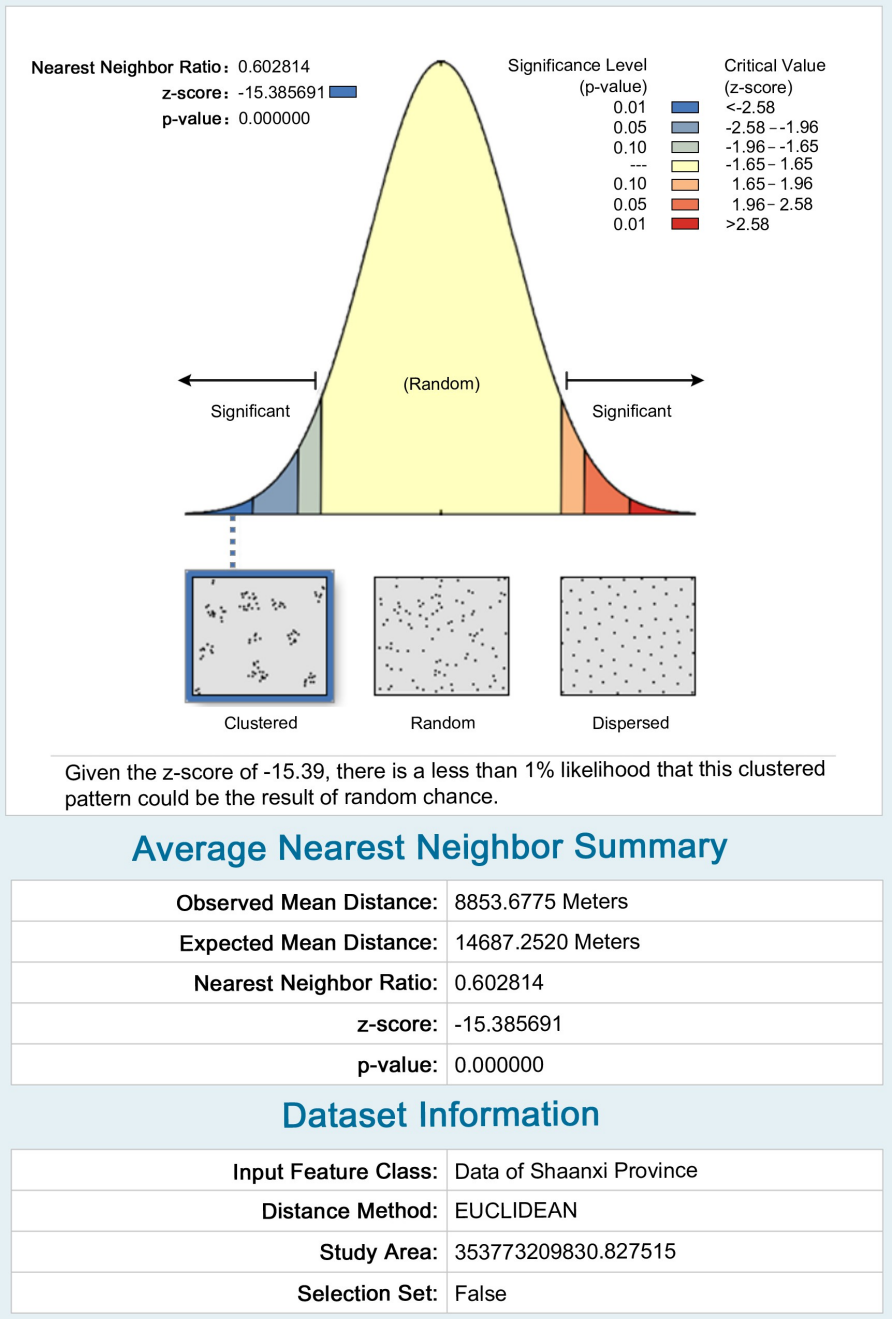
Nearest neighbor index analysis chart.

The spatial relationships can be broadly classified into three patterns: aggregated, dispersed, and random. According to the results of the nearest neighbor index, it can be seen that the nearest neighbor ratio of the fort-type settlements in Shaanxi is less than 1, the p-value is 0, and the z-score is much lower than 0, which prove that these data are significantly aggregated (Fig 5).

**Fig 6:**
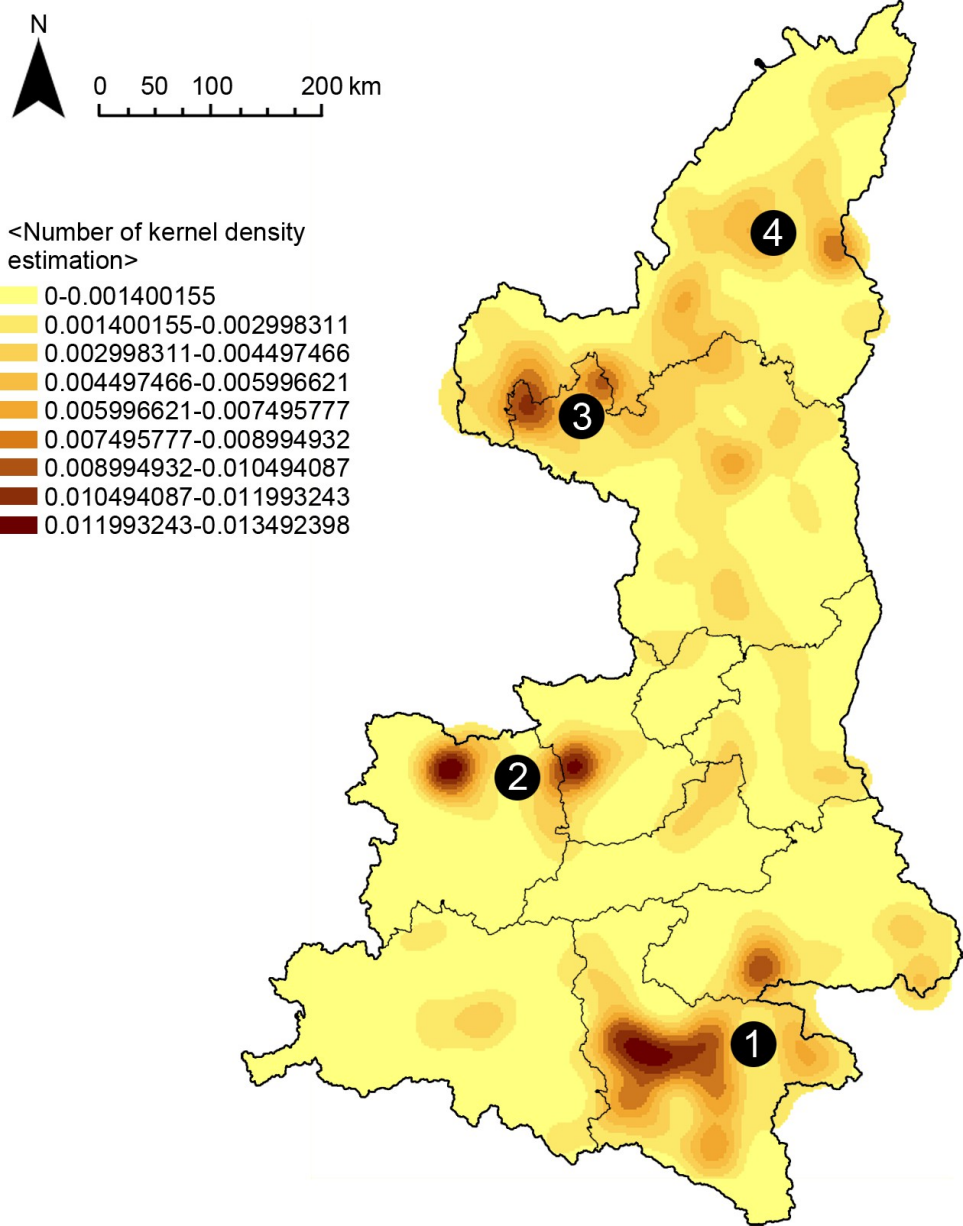
Kernel density estimation map.

Kernel density analysis was conducted on the data of fort-type settlements, and according to the kernel density estimation results, it can be seen that the fort-type settlements in Shaanxi are aggregated, indicating that the fort-type settlements in this area form multiple cluster systems under the influence of each variable. There are four clustering centers, and the first three clustering areas show a point-like clustering distribution, while the fourth one shows a linear clustering distribution (Fig 6).

### Superposition of spatial and non-spatial properties

**Fig 7:**
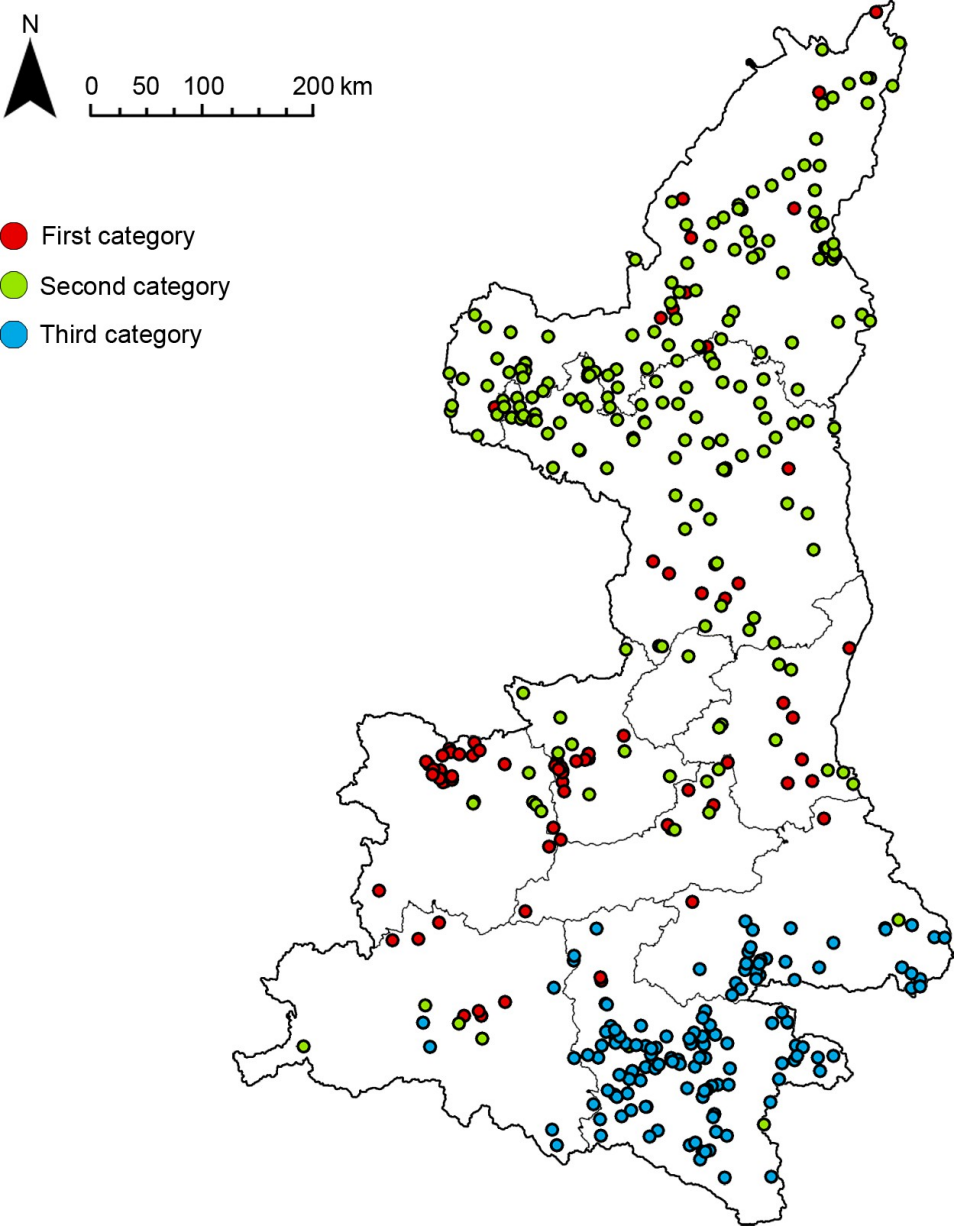
Cluster distribution map.

The results of the cluster analysis show that the fort-type settlements clusters in the Shaanxi region could be classified into three main types by different attributes. In order to understand the spatial characteristics of these data, the three types of data derived from the cluster analysis were imported into ArcGIS and their aggregation was observed (Fig 7). The results show that the three types of fort-type settlements not only produce aggregation effects in two dimensions but also show aggregation characteristics in terms of geographical distribution, which are located in the south, central, and north of Shaanxi Province.

**Fig 8:**
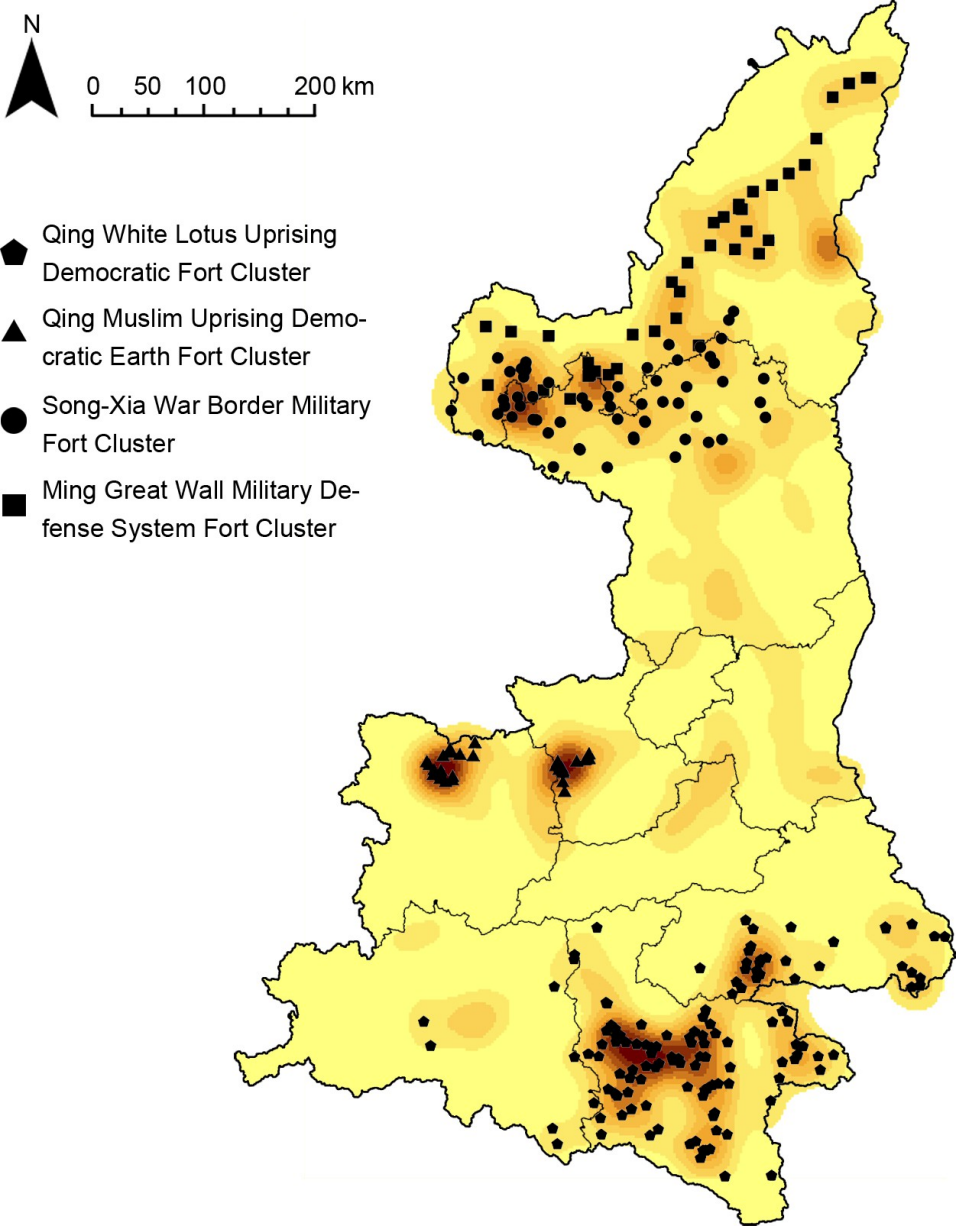
Group system distribution map.

The results of cluster analysis and density analysis are highly correlated. By overlaying the results of cluster analysis with the results of kernel density analysis, the final clusters of fort-type settlements would have attributes of both spatial and category dimensions, generating a geospatial cluster system (Fig 8). The three types of fort-type settlements data were analyzed and compared geospatially, and four fort-type agglomerations with distinct characteristics were finally found.

### Reasons and influencing factors of cluster formation based on correlation analysis

**Fig 9:**
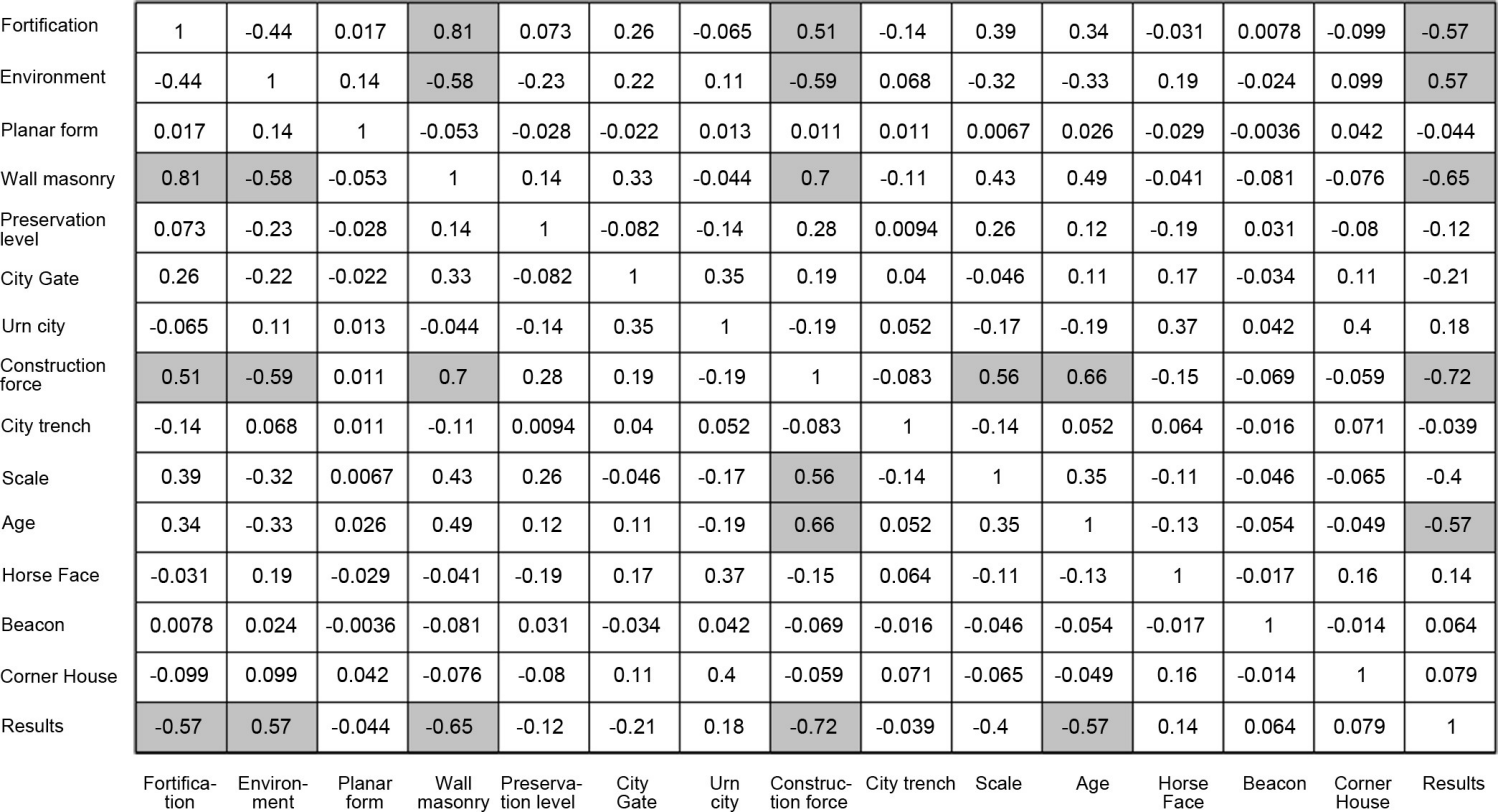
Correlation analysis.

What factors lead to the differences between the different cluster types? Based on this query, this paper adopted the correlation analysis method to study the overall data. The correlation analysis between the influencing factors and the clusters shows (Fig 9) that the factors that have the greatest influence on the clustering results are, in order, construction force (0.72), wall masonry (0.65), age (0.57), fortification purpose (0.57), and topographic environment (0.57). Among the five main influencing factors, construction force, age, and fortification purpose are historical and social attributes, the topographic environment is a natural geographic attribute, and wall masonry is an architectural attribute, which proves that all three attribute dimensions have an important influence on the distribution of fort-type settlements, and also prove the rationality and validity of the division of influencing factors.

**Fig 10:**
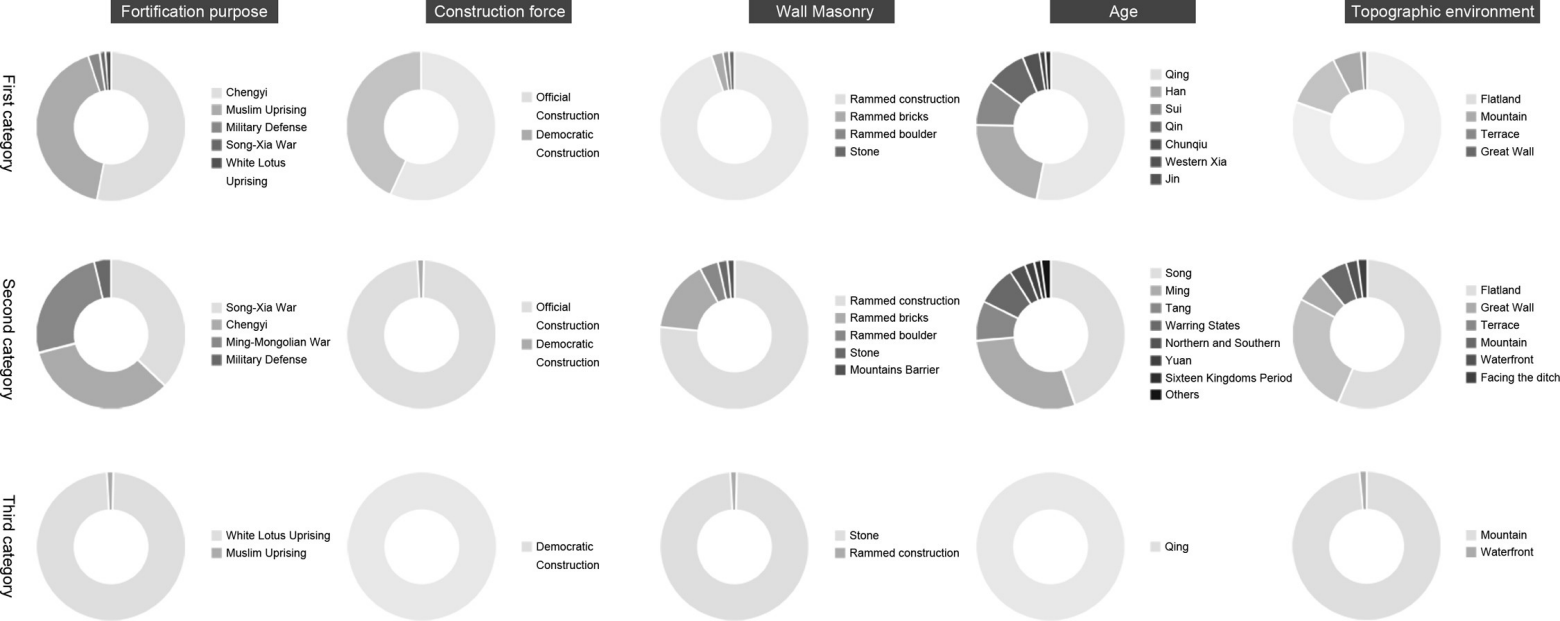
Type factors statistical chart.

The proportion of the five influencing factors in the three types of forts were analyzed statistically separately, and the characteristics of each of the three types can be inferred (Fig 10). The characteristics related to the third type of forts are clearer, with the main distribution dating from the Qing Dynasty, the construction force being civil society, the defense purpose being the White Lotus uprising, the wall masonry being mainly stone, and the terrain environment being mountainous. The characteristics of the first and second types of forts are mixed and not obvious. The first category mainly includes the Qing and Han dynasties, and the purpose of fortification accounts for the largest proportion of the Muslim uprising and Chengyi, and the proportion of the two types of government construction and civil construction is close, so we can initially judge the existence of two subtypes within. The second category mainly includes the Song and Ming dynasties, and the largest proportion of fortification purposes are the Song-Xia War, Chengyi, and the Ming-Mongolian War, so it is assumed that there are two or three subtypes within.

Combining the four group systems derived in the previous section, it is clear that the first cluster system belongs to type III, the second cluster system belongs to type I, and the third and fourth cluster systems both belong to type II. When the data of the four clusters are analyzed and compared, it is found that there is a high degree of consistency within the clusters, while the differences between the clusters are large. Based on the attributes of the five influencing factors that have the greatest impact on the distribution of the clusters, the four clusters are named the Qing White Lotus Uprising Democratic Fort Cluster, the Qing Muslim Uprising Democratic Earth Fort Cluster, the Song-Xia War Border Military Fort Cluster, and the Ming Great Wall Military Defense System Fort Cluster. Then the effects of each of the five factors on the distribution of the fort-type clusters are analyzed below.

#### Construction force

**Fig 11:**
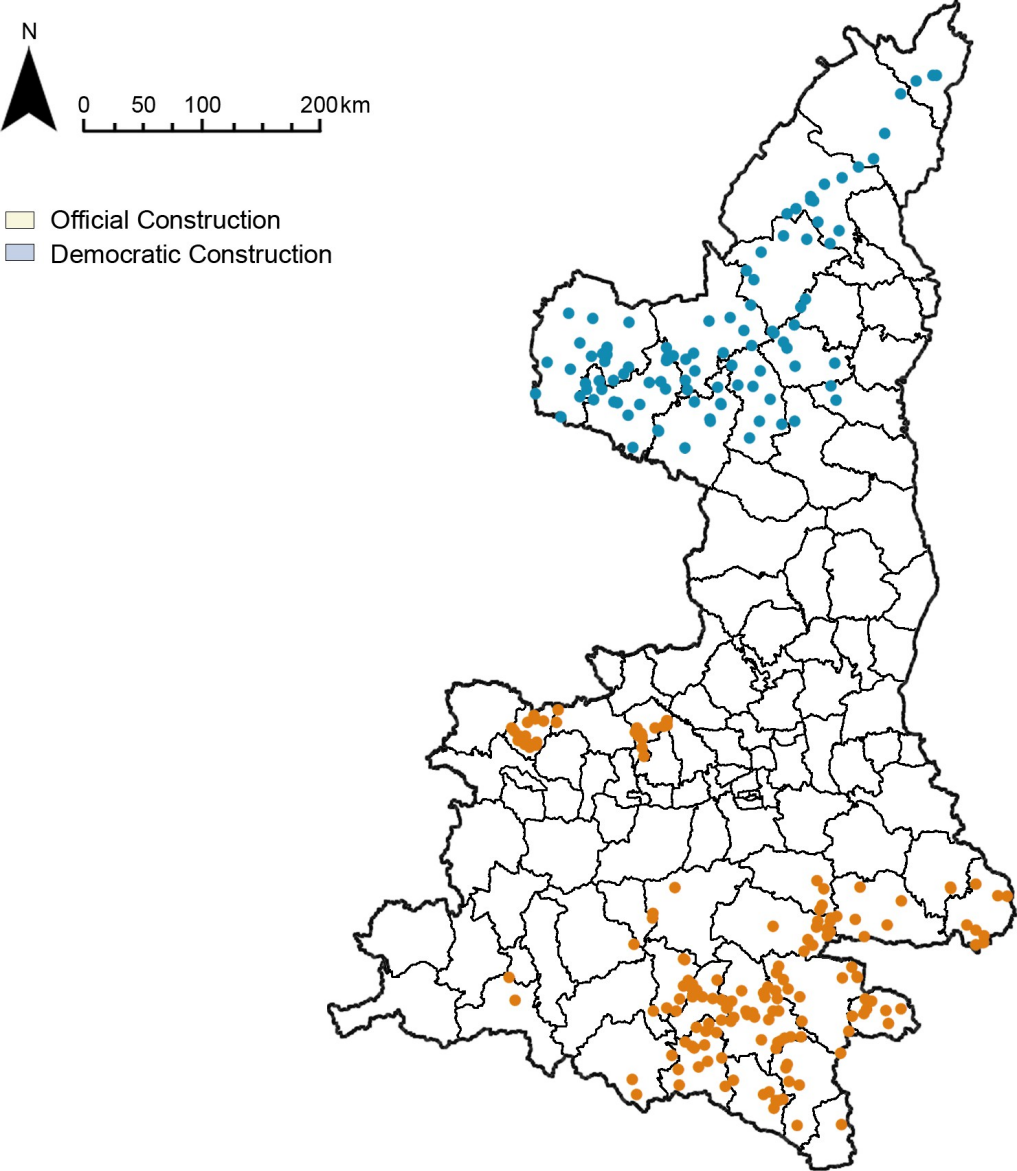
Distribution map of different construction forces.

Man-made unstable factors are generally regarded as the original cause of fort-type settlements, including war, mutiny, armed struggle, banditry, etc., among which border wars and interior wars are the two main factors leading to the construction of fort-type settlements on a large scale [25]. The former mainly refers to the wars between the Central Plains dynasty and the neighboring minority groups in the frontier areas and is the direct cause of the formation of the cluster system of government-built fort-type settlements. The latter mainly refers to the struggles located in the internal hinterland of China, and have an important influence on the construction of civilian fort-type settlements. The objective results of the distribution of the four cluster systems can support this view. Government-built fort-type settlements such as the Song-Xia War Border Military Fort Cluster and the Ming Great Wall Military Defense System Fort Cluster are located in northern Shaanxi, which is the border area during Song and Ming dynasties. While civilian fort-type settlements such as the Qing White Lotus Uprising Democratic Fort Cluster and the Qing Muslim Uprising Democratic Earth Fort Cluster are located in the central and southern hinterland of Shaanxi (Fig 11).

#### Fortification purpose

**Fig 12:**
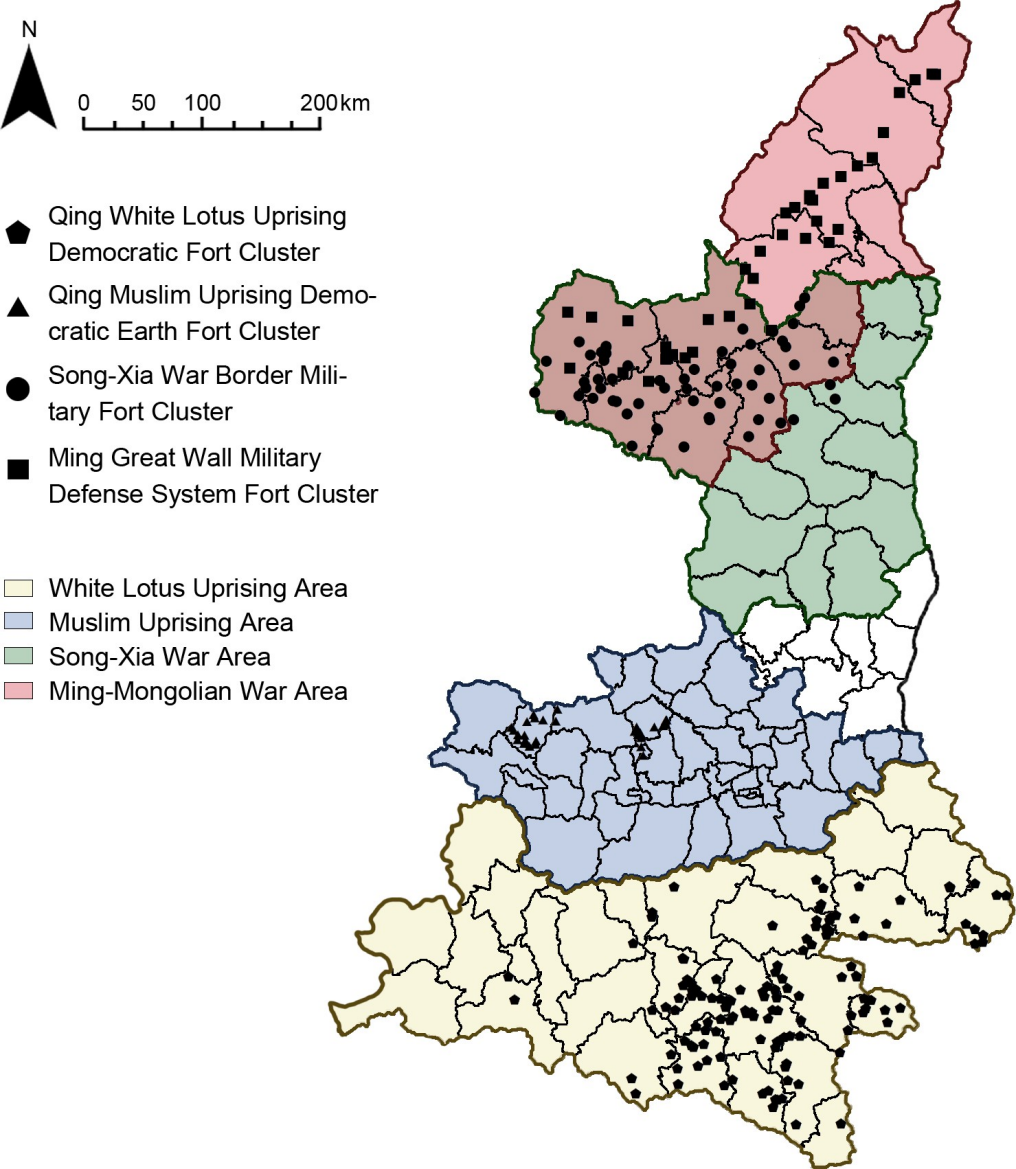
Scope of the war conflict.

Based on historical documents, we studied the impact areas of historical conflicts and wars in Shaanxi [26–29] and found that the distribution areas of the four clusters overlap with the impact areas of White lotus uprising, Muslim uprising, Song-Xia war, and Ming-Mongolian war in historical records (Fig 12). It proves that the "fortification purpose" factor has an important influence on the distribution of settlements. Generally speaking, the "fortification purpose" factor is directly responsible for the construction of fort-type clusters.

#### Wall masonry

Wall masonry is the material required for the construction of fort-type settlements and is closely related to the geographical environment in which the settlements are located. The correlation coefficient between wall masonry and topographic environmental is -0.58, which proves that they have a strong correlation. The different wall masonry factors lead to the different external morphology of the fort-type clusters. As mentioned in *Renewed Shaanxi General Records Draft*, "fort-type settlements differed from those of high mountains and flat lands" [30]. The walls of rammed fort-type settlements are heavy and easily eroded by wind and rain. Most of the stone forts are located in mountainous areas, so it is easy to get materials from local sources. Due to the characteristics of stone, fort-type settlements constructed by stone are preserved longer.

#### Age

**Fig 13:**
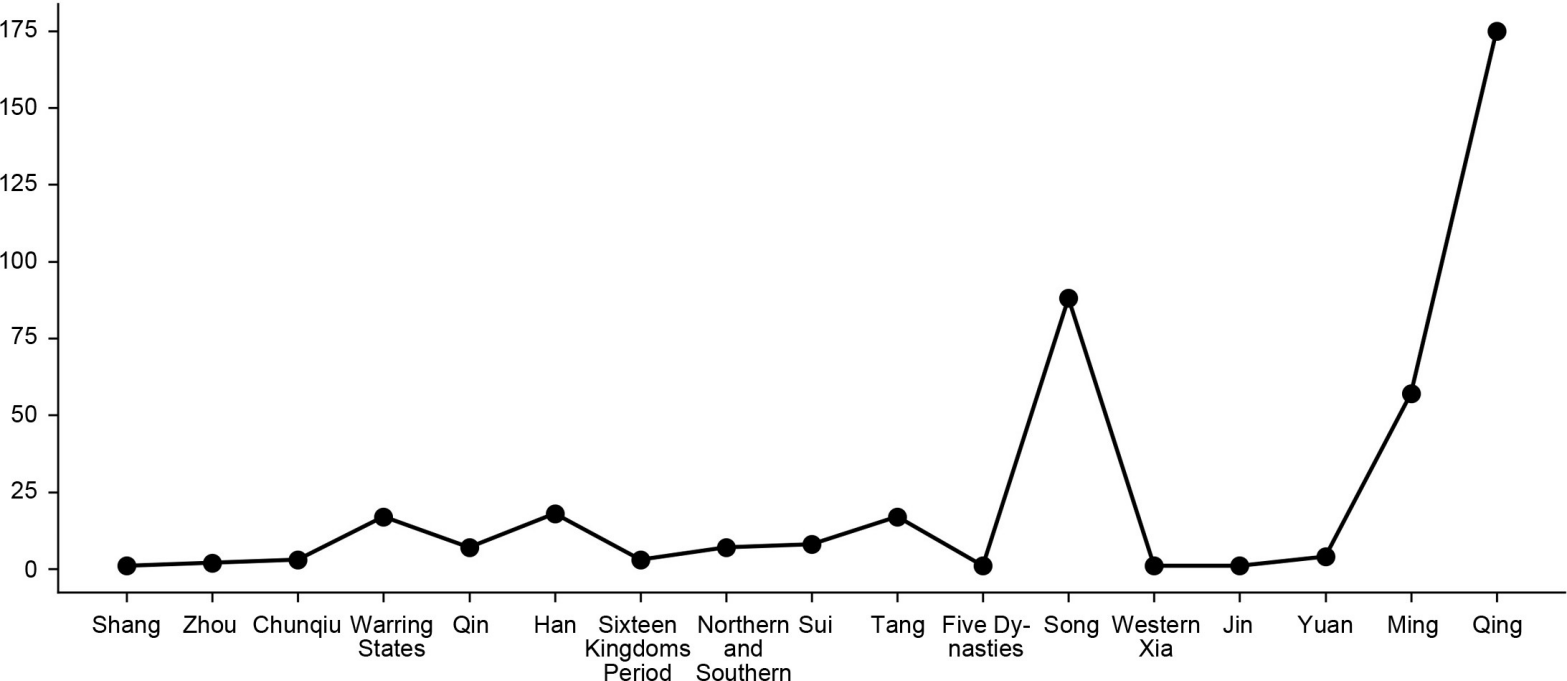
The chronological trend of fort-type settlements.

An overall chronological trend analysis of the fort-type settlements in Shaanxi (Fig 13) reveals that the construction of fort-type settlements shows a significant increase in number during the Song, Ming, and Qing dynasties, which coincides with the chronology of the cluster system. The influence of age on the fort-type cluster is twofold: Firstly, many surviving fort-type settlements date back to the era when the largest number of fort-type settlements are built-in history, due to the numerical advantage. Secondly, the fort-type settlements of different periods are influenced by the times and show different characteristics. In the Song Dynasty, the development of crossbows, guns, and gunpowder led to the development of a deep defense system, and multiple walls and trenches were built to increase the strength of the defense. During the Ming Dynasty, bricks began to be used on a large scale in above-ground construction, so a large number of rammed and brick-clad fort-type settlements began to appear. In the Qing Dynasty, the government encouraged people to defend themselves, which led to the large-scale construction of civilian fort-type settlements.

#### Topographic environment

**Fig 14:**
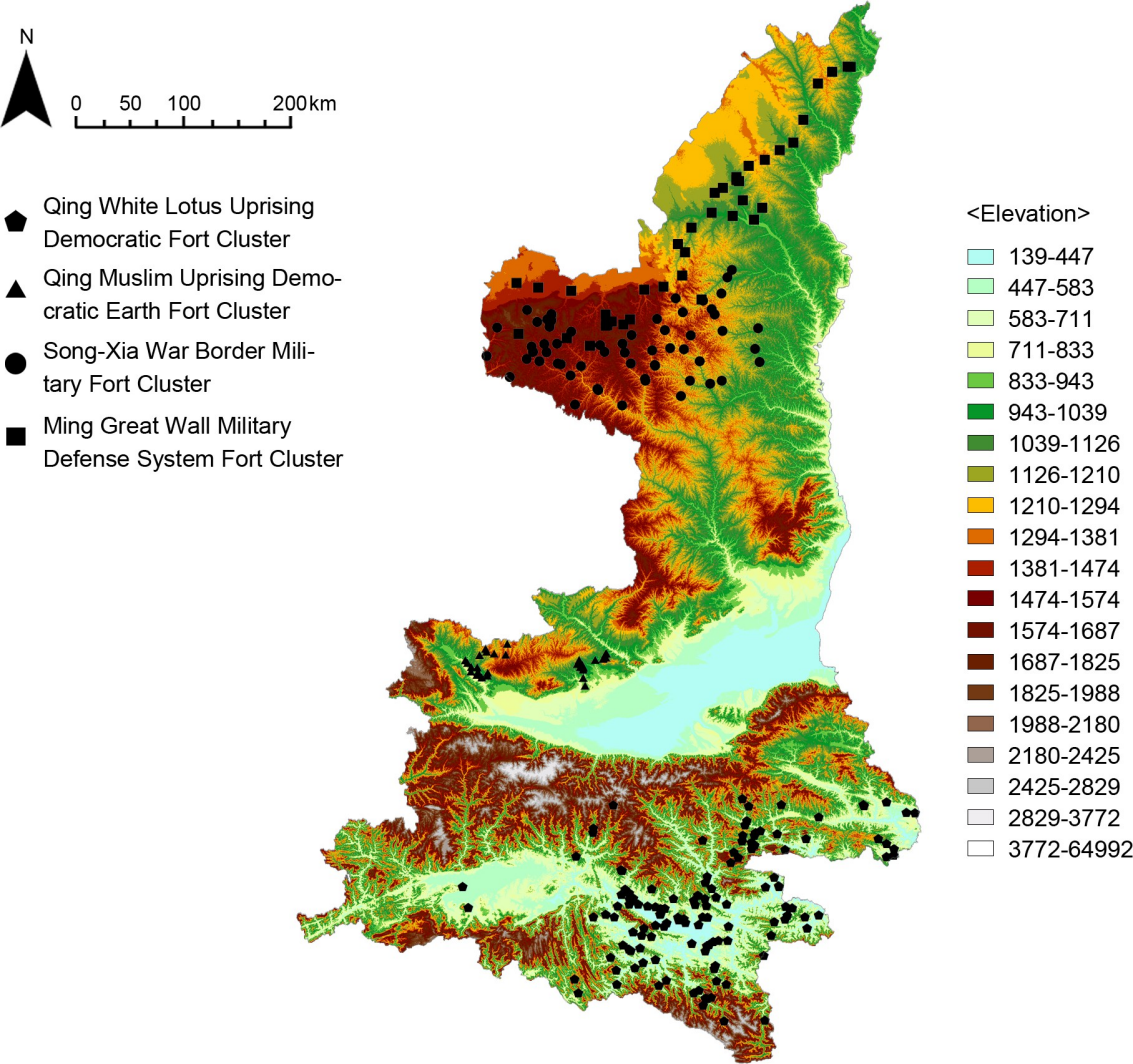
Analysis of elevation.

**Fig 15:**
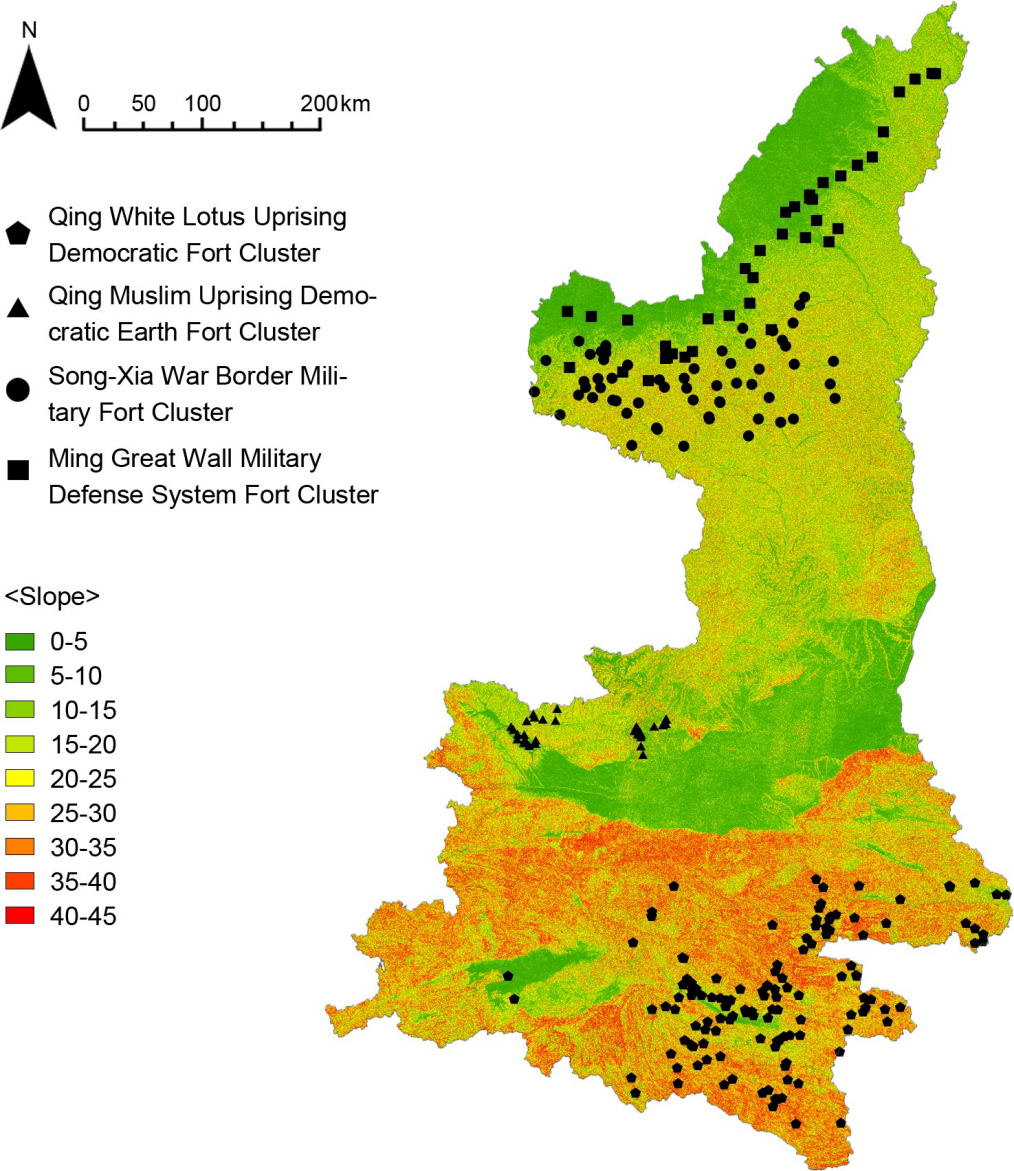
Analysis of slope.

**Fig 16:**
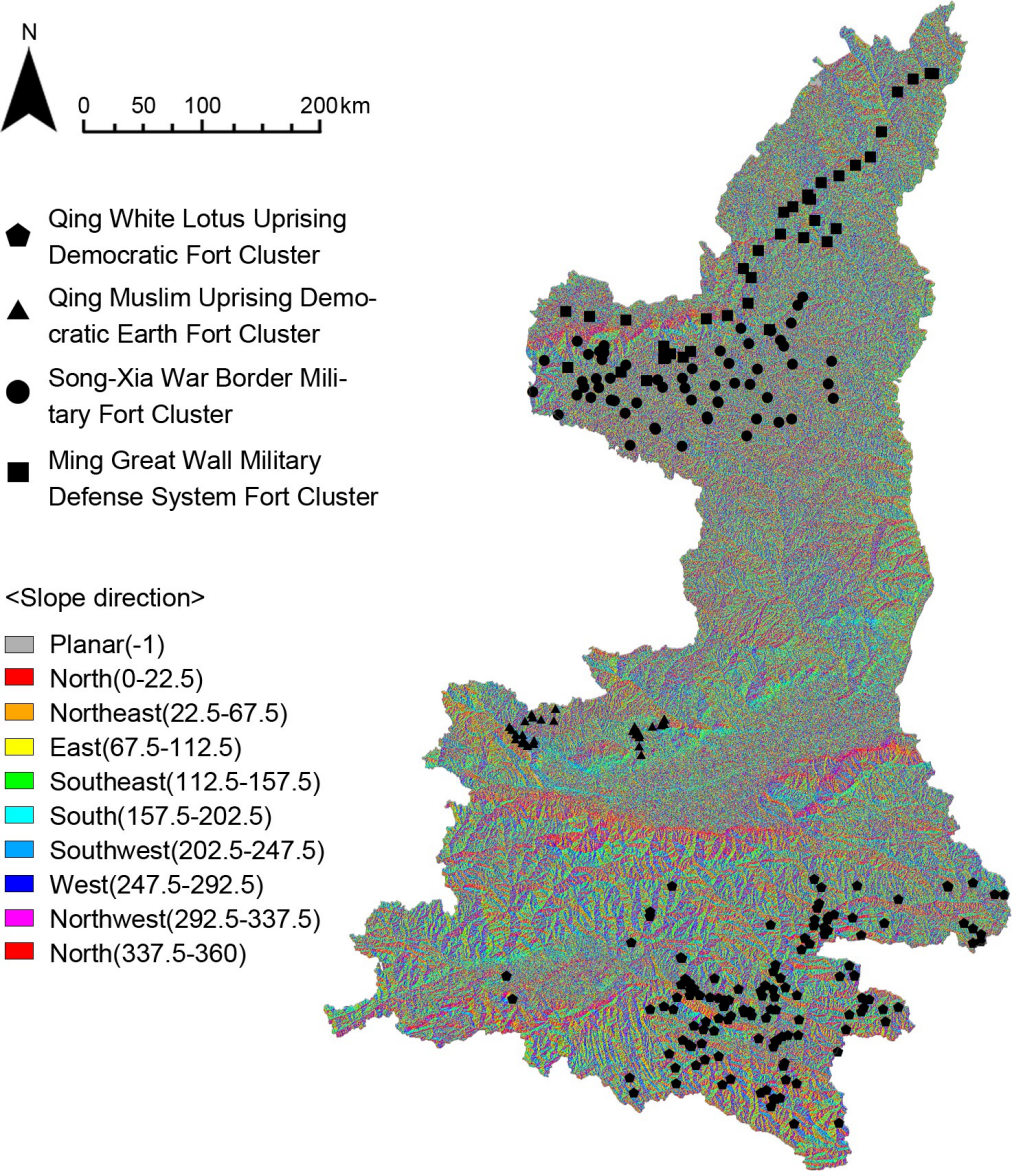
Analysis of slope direction.

**Fig 17:**
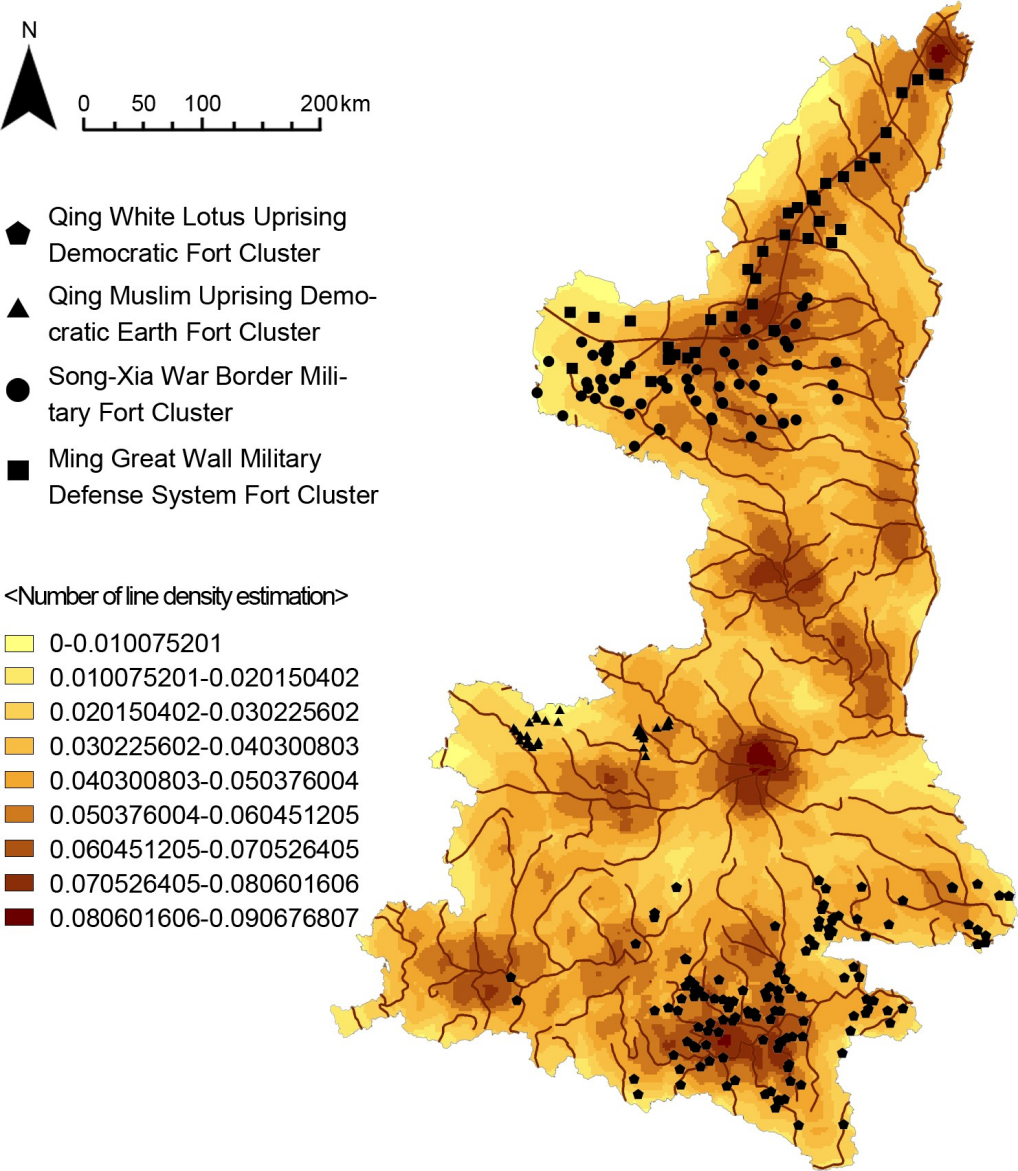
Line density map.

The physical geography of the Shaanxi region was analyzed by GIS in order to study the influence of topographic environmental factors on the distribution of the fort-type clusters, including DEM digital elevation model, slope analysis, slope direction analysis, and line density analysis of the Great Wall and rivers. The results show that (Figs 14–16) the Qing White Lotus Uprising Democratic Fort Cluster is distributed in a complete geographic unit (Qinba Mountains), where the slope is highest. The Qing Muslim Uprising Democratic Earth Fort Cluster is distributed in central Shaanxi, with a relatively low slope. The other two clusters are distributed in the northern plains of Shaanxi, and the distribution areas overlap. The Song-Xia War Border Military Fort Cluster is concentrated in the higher elevation area, while the Ming Great Wall Military Defense System Fort Cluster shows an obvious linear distribution along the Ming Great Wall (Fig 17). It proves that fort-type clusters are usually formed within a complete geographical unit. The reason is that mountain and water systems act as a natural barrier to cultural migration and transmission [31].

**Fig 18:**
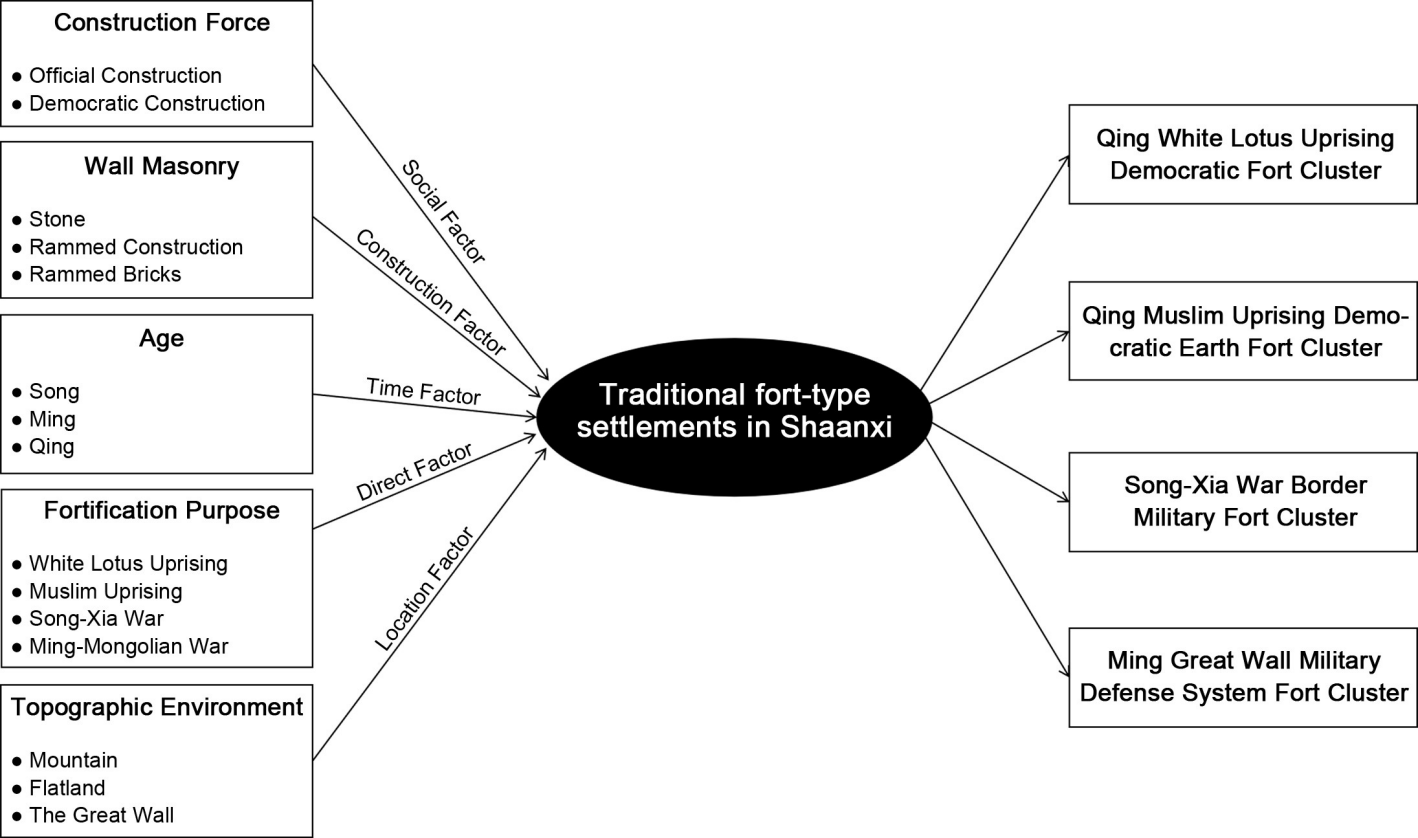
Influencing factors action process.

To sum up, among the five influencing factors, the purpose of fortification is the direct factor, representing the specific conflict events that prompted the construction of the fort-type clusters. And age, topographic environment, construction force, wall masonry are indirect factors, representing the time, place, subject, and construction of the conflict event, respectively (Fig 18). These factors have a multifaceted and complex impact on the fort-type settlements, ultimately leading to the formation of the cluster system in Shaanxi and the differences between clusters. These influencing factors do not need to act simultaneously. For example, the Qing White Lotus Uprising Democratic Fort Cluster and the Qing Muslim Uprising Democratic Earth Fort Cluster are both fort-type groups in the context of folk uprisings in the Qing Dynasty, but they have completely different forms due to the differences in topographical environment and construction materials. The Song-Xia War Border Military Fort Cluster and the Ming Great Wall Military Defense System Fort Cluster are both built in the area of northern Shaanxi, but the differences in military systems between the Song and Ming governments lead to differences in the character of them.

### Characteristics of the different cluster systems in Shaanxi

The analysis of the nearest neighbor index (Table 2) was conducted on the four cluster systems in Shaanxi. And it is found that the p-values of all cluster systems are close to 0 and the nearest neighbor index is less than 1. The smaller the ANN index, the higher the degree of clustering. It indicates that all cluster systems have aggregated distribution characteristics, which also proves the rationality and validity of the previous division. A comparative study of the four cluster systems was conducted to analyze their respective characteristics (Table 3).

**Table 3:** Characteristics of cluster systems classification.

Impact factors	Qing White Lotus Uprising democratic fort cluster	Qing Muslim Uprising democratic earth fort cluster	Song-Xia War border military fort cluster	Ming Great Wall military defense system fort cluster
Age	Qing	Qing	Song	Ming
Fortification purpose	The White Lotus Uprising	Muslim Uprising	Song-Xia War	Ming-Mongolian War
Construction force	Democratic Construction	Democratic Construction	Official Construction	Official Construction
Cultural preservation level	low	low	Medium	high
Topographic environment	Mountain	Flatland	Flatland	The Great Wall
Planar form	Rectangular and Square predominate,with the rest including irregular shapes, circles and squares.	Rectangular	Rectangular shapes predominate, with the rest including trapezoids, ovals and irregular shapes.	Rectangular
		Square		Square
Wall masonry	stone	rammed construction	mainly by rammed construction	Rammed construction Rammed bricks
Scale	5001–100,000 m^2^	5001–100,000 m^2^	10001–1000,000 m^2^	10001–1000,000 m^2^

#### Qing White Lotus uprising democratic fort cluster

The fort-type cluster was located in the mountainous region of southern Shaanxi and was fortified in the context of the White Lotus uprising in the mid-Qing period. The White Lotus uprising started in Hubei and was more severely affected in Sichuan, Shaanxi, and Gansu, while Henan and Hunan were only partially affected, which is why it is also known as the "Great Peasant Uprising in the Five Provinces of Sichuan, Chu, Shaanxi, Gansu, and Henan" [32]. The southern part of Shaanxi province was the main battleground for the activities of the White Lotus sect because of its mountainous terrain, so a large number of fort-type settlements were built for refuge. The forts were built during the Qing Dynasty and had a low level of cultural preservation. Because of the abbreviated construction method, the fort-type settlements as a whole are severely damaged, with only stone walls and buildings surviving, and almost no ancillary defense facilities such as horse faces and corner towers. The main features are: Firstly, the fort-type settlements are located on top of high mountains, and extremely secretive, easy to defend, and difficult to attack. The cliff walls are often used as barriers, forming a defense form of "steep on three sides and passable on one side" [33]. The fortification gate is cut off, and a boarding bridge is placed to pass through when nothing happened, and the boarding bridge is removed when the bandits come to invade. Secondly, the material of the fort-type settlements is all stone. It is built on the mountain, so the material is taken from the local area and the settlements are built with stone. Thirdly, influenced by the mountain situation, the form of the fort-type settlements is mainly rectangular, round, and irregular. Fourthly, most of the forts are built by the private sector, which has limited human and financial resources, so the scale is small, and most of the forts are built by several families together.

#### Qing Muslim uprising democratic earth fort cluster

These fort-type settlements were mainly located in central Shaanxi and were built under the influence of the Hui rebellion during the Tongzhi period of the Qing Dynasty. In this situation, people built forts to protect themselves. Statistics show that these fort-type settlement clusters are dated to the Qing Dynasty, with a low level of cultural protection and extremely simple architectural forms. The shape is mainly rectangular and square, and most of them have only one gate, no horse face, corner tower, and other ancillary defense facilities. The main features are: Firstly, the majority of the settlements are built on flat ground, called "Tu Wei Zi". Historical records show that in the northwest of the country, there are "many large surnames, all of whom lived together and built fort-type settlements" [34]. Secondly, the forts are mostly built by civil engineering, and the defense facilities are simple and with folk characteristics. The settlements form is not quite the same as the Central Plains, the wall is high and heavy, "the door is small and high, there are also the tall building" [35]. Its defense facilities are usually relatively simple, outside a circle of the fence and a female wall, no trenches, and forts, generally in the corner of the Zhuang or near the gate with a high room, used to look out for the enemy. Thirdly, the function of the fort-type settlements is mainly residential. There are also large-scale forts with two cities, an inner city is a place of residence and an outer city is a place of production and labor such as workshops, vegetable beds, and livestock pens.

#### Song-Xia War border military fort cluster

The formation of this type of fort-type settlements system was mainly influenced by the Song-Xia War, which was distributed in northern Shaanxi. Since the founding of the dynasty, the conflict between Song, Liao, and Western Xia in the northwest region almost never stopped, among which the Song-Xia War was the most frequent. In order to guard the frontier, the Northern Song Dynasty set up troops for long-term defense in northern Shaanxi, "building cities due to the phase terrain, exhausting the key places, and setting up fort-type settlements with soldiers" [36]. These fort-type settlements survive at present, and are also known as "Song Xia Fortress". Only some of the settlements are well preserved, while the rest only have remnants of walls, and most of the defensive facilities such as gates, trenches, piers, and horse faces are not present. The main features are: Firstly, the form is mainly rectangular, often oval, trapezoidal, irregular, and other forms. Because of the complex topographic environment, including mountains, terraces, ditches, and other terrains, fort-type settlements are built according to the terrain and often show irregular forms. Secondly, there are double cities or back-shaped cities. A double fort-type settlement is also known as a "double-clothed fort-type settlement", where two fort-type settlements are built next to each other or even share a common wall. Thirdly, the internal functions of the fort-type settlements include administration, storage, military, and residence, while the military training ground, martial arts pavilion, horse farm, and other functional areas are permanently located outside the settlements.

#### Ming Great Wall military defense system fort cluster

These forts were built by the imperial court in the context of the Ming-Mongolian War and were mainly located in northern Shaanxi. In order to defend against nomadic invasion, the Ming Dynasty built the Great Wall and a series of fortifications along the northern frontier. These military fort-type settlements were built by the state, with different levels of road towns, guard towns, and fortress towns, forming a tight military defense system. The settlement group is concentrated in the Ming Dynasty, and the proportion of provincial, municipal and county-level cultural protection level fortresses tends to be nearly half, with a high cultural protection level. But most of the defense facilities such as urn cities, trenches, horse faces, and corner towers have been destroyed. The main features are: Firstly, the forts are built in accordance with the Ming Great Wall and are linearly distributed. During the Ming Dynasty, nine major towns were established to defend against hostile forces, and most of the Ming military fort-type settlements in present-day Shaanxi Province belong to the Yulin town fortress system, so the clusters are generally distributed along the Ming Great Wall. Secondly, the settlement form is relatively regular, mainly rectangular and square, often in the form of central symmetry. Thirdly, the material of the fort-type settlements is mainly rammed and wrapped bricks. Fourthly, the internal functions include military defense, economic trade, production, and residence, etc.

## Conclusion

This paper introduces clustering algorithm, correlation analysis, principal component analysis, kernel density estimation, and nearest neighbor index to investigate the clusters and formation factors of fort-type settlements in Shaanxi. The conclusions are as follows.

1. The results of the K-means clustering algorithm show that the fort-type settlements clusters in Shaanxi can be divided into three types according to their attributes, and their main characteristics are taken as the basis for identification.
2. Four clustered fort-type settlements are found in Shaanxi, which locate in the southern, central, and northern border areas of Shaanxi, respectively. The forts fortified for the White Lotus Revolt, the Muslim Revolt, the Song-Xia War, and the Ming-Mongolian War are highly aggregated, while the forts fortified for city and military defense do not have obvious aggregation characteristics.
3. There are four typical fort-type settlements clusters in Shaanxi, namely, Qing White Lotus Uprising Democratic Fort Cluster, the Qing Muslim Uprising Democratic Earth Fort Cluster, the Song-Xia War Border Military Fort Cluster, and the Ming Great Wall Military Defense System Fort Cluster. These settlements have strong internal consistency and external variability, forming a distinctive fortress cluster system.
4. The results of correlation analysis show that these clusters are formed mainly by the construction force, wall masonry, age, fortification purpose, and topographic environment factors.

The quantitative research methods of spatial distribution, cluster system division, and formation causes of fort-type settlements have proved to be effective and can be applied to other studies related to settlements. At the same time, the perspective of cluster systems has important theoretical and applied values. Firstly, in heritage conservation, the government can introduce a cluster-based conservation model. There are a large number of internally related fort-type settlements in China, but their cultural preservation units have not formed a good interoperability system. And some of the existing civilian fort-type settlements have a low level of heritage protection, which are easily neglected and destroyed. The "cluster system" is the ideal form of heritage conservation for the whole of the fort-type settlements in the region, which can build a genealogy of fort-type settlements heritage based on "Age-Environment-Fortification purpose-Construction force-Wall masonry" and can avoid the omission of individual forts to a greater extent. Secondly, the relevant departments can use the cluster system for internal restoration and speculation, the common characteristics of the cluster can serve as a counter-inference to the study of the individual forts. A large number of surviving fort-type settlements have no relevant historical records, and their historical situation can be inferred from similar settlements within the same group distribution area. At the same time, if the causes, locations, and dates of the construction of certainly damaged forts are known, their architectural forms can be reasonably inferred from other existing settlement forms within the same group, which provides theoretical support for the restoration of heritage settlements. Thirdly, it is recommended that policymakers should not only study architecture but also pay more attention to the historical background and social organization of the clusters because human-historical factors are more influential in the formation of fort-type clusters.

In addition, during the process of data collection and cleaning, it was found that there were many fort-type settlements with missing information in various aspects such as age, fortification purpose, and the number of defense facilities, which made it extremely difficult to enter and classify data information and was also not conducive to the subsequent heritage conservation. Theoretically, fort-type settlements with temporarily missing information should not be excluded from the cluster system, which is a shortcoming of this study. Therefore, how to use the information of existing fort-type settlements to build a prediction model, which can include fort-type settlements with missing information as well as undiscovered fort-type settlements in the relevant cluster system, will become the focus of subsequent research.

## Supporting information

S1 File Analysis of the spatial distribution of fort-type settlements in Shaanxi. (ZIP)

S2 File Clustering analysis line chart. (TIF)

S3 File Clustering analysis scatters plot. (TIF)

S4 File Nearest neighbor index analysis chart. (ZIP)

S5 File Correlation analysis. (TIF)

S6 File Type factors statistical chart. (TIF)

S7 File The chronological trend of fort-type settlements in Shaanxi. (TIF)
