# Supplementary figures and images for: Research on cluster system distribution of traditional fort-type settlements in Shaanxi based on K-means clustering algorithm
Source: PLoS One. 2022 Mar 11;17(3):e0264238. doi: 10.1371/journal.pone.0264238 (PMC8916632; doi:10.1371/journal.pone.0264238)

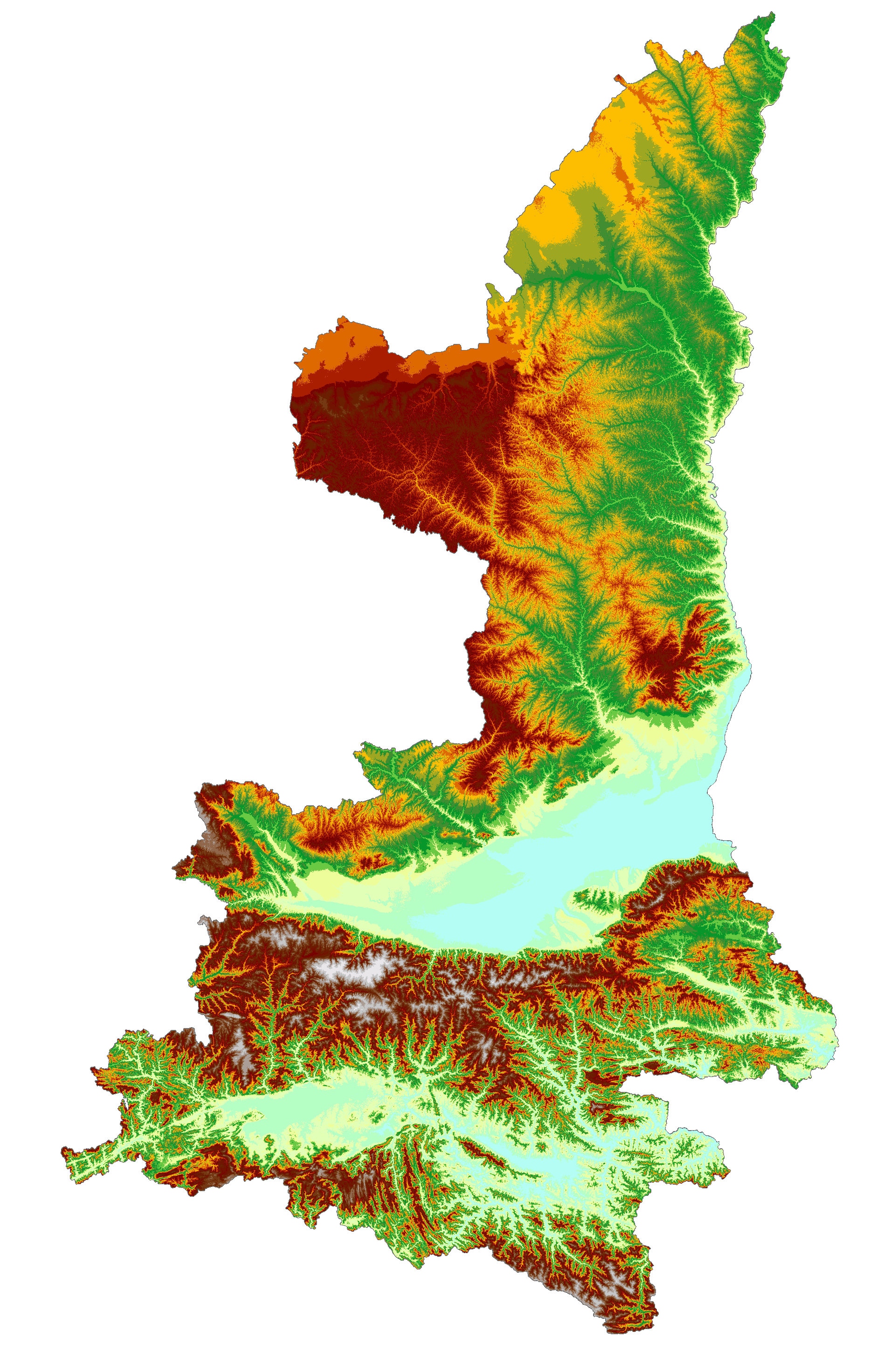

Supplement: S1 File — (ZIP) [file pone.0264238.s001.zip › S1 File. Analysis of the spatial distribution of fort-type settlements in Shaanxi/Analysis of elevation.tif]

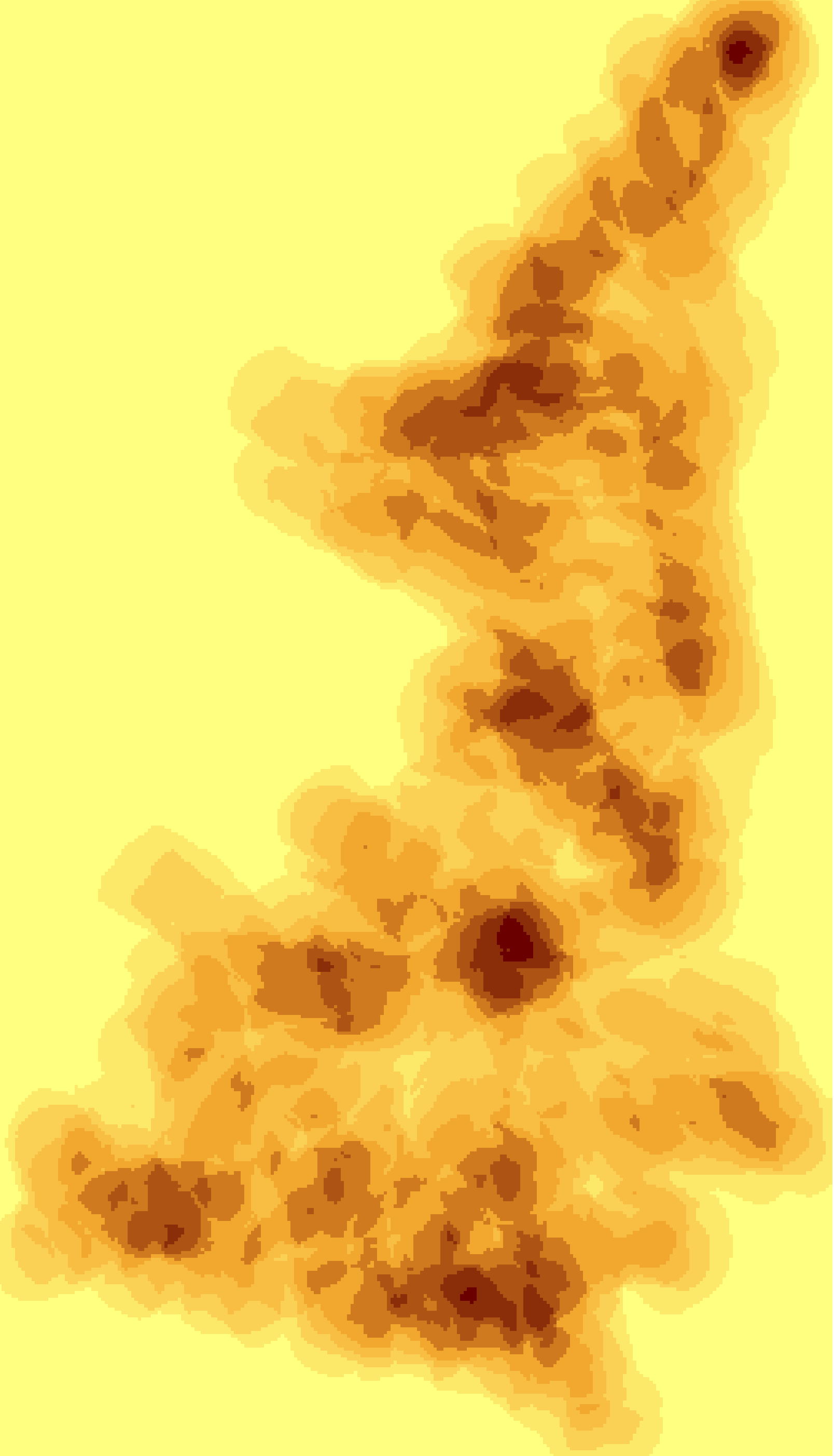

Supplement: S1 File — (ZIP) [file pone.0264238.s001.zip › S1 File. Analysis of the spatial distribution of fort-type settlements in Shaanxi/Analysis of Line Density.tif]

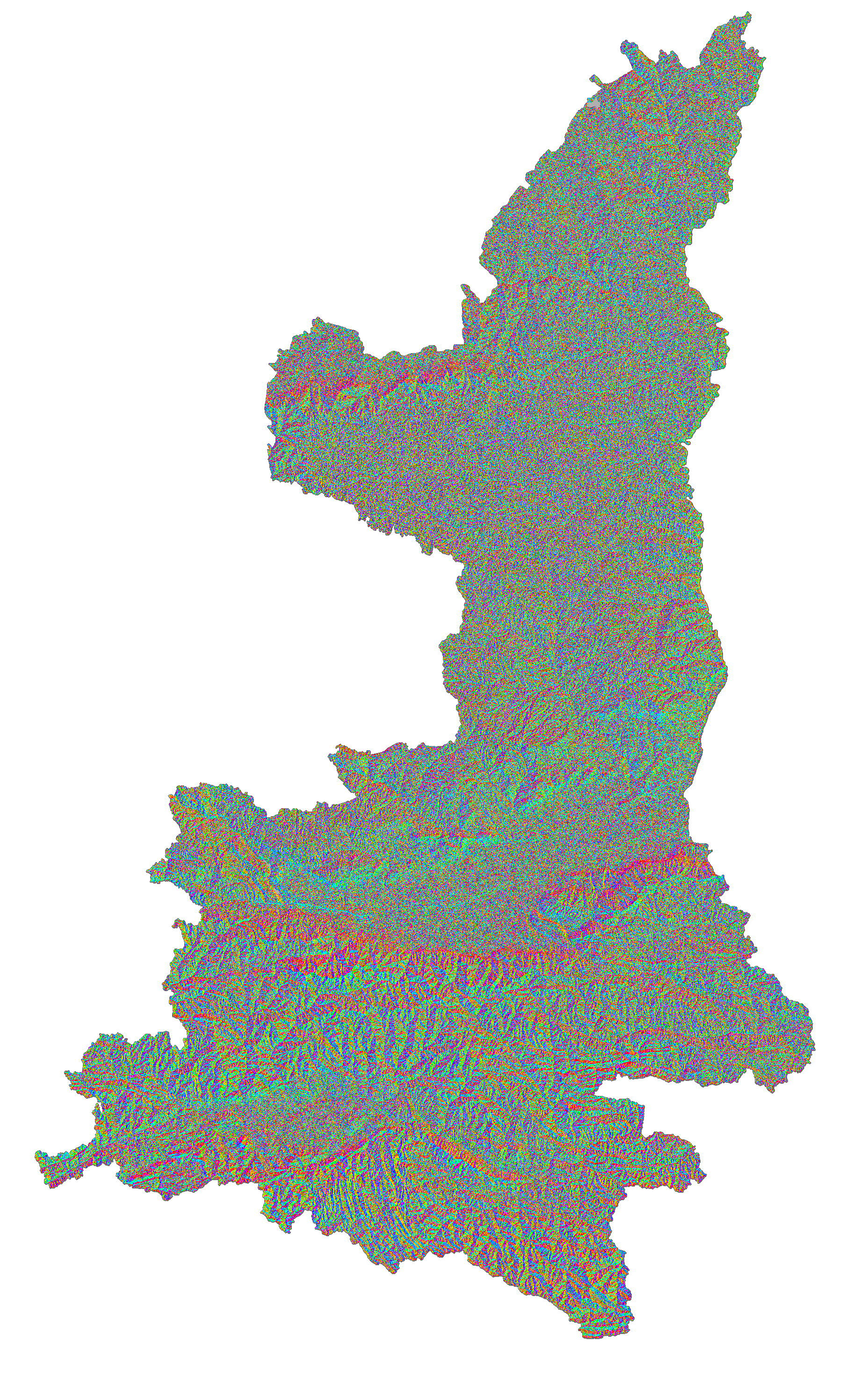

Supplement: S1 File — (ZIP) [file pone.0264238.s001.zip › S1 File. Analysis of the spatial distribution of fort-type settlements in Shaanxi/Analysis of slope direction.tif]

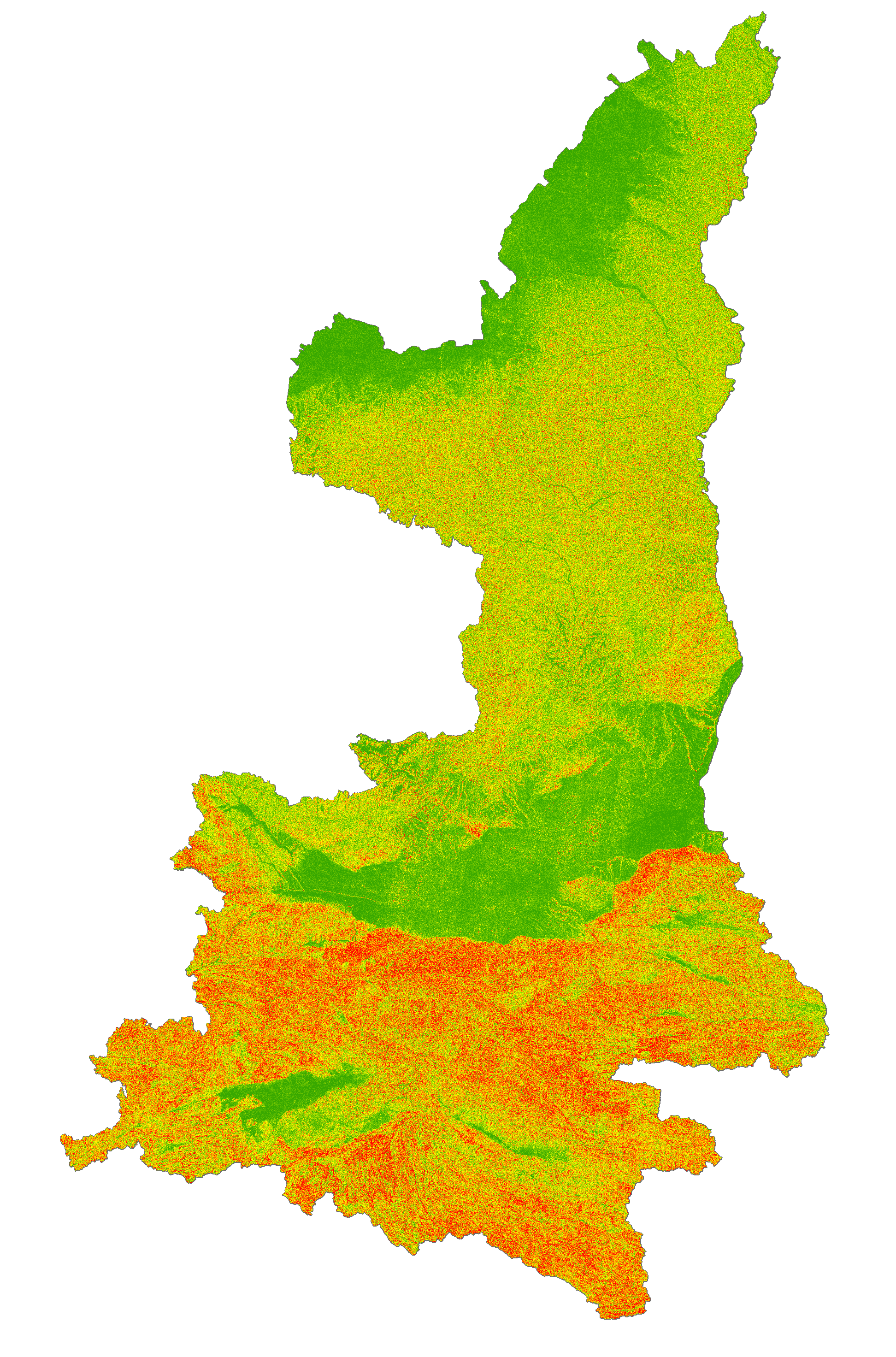

Supplement: S1 File — (ZIP) [file pone.0264238.s001.zip › S1 File. Analysis of the spatial distribution of fort-type settlements in Shaanxi/Analysis of slope.tif]

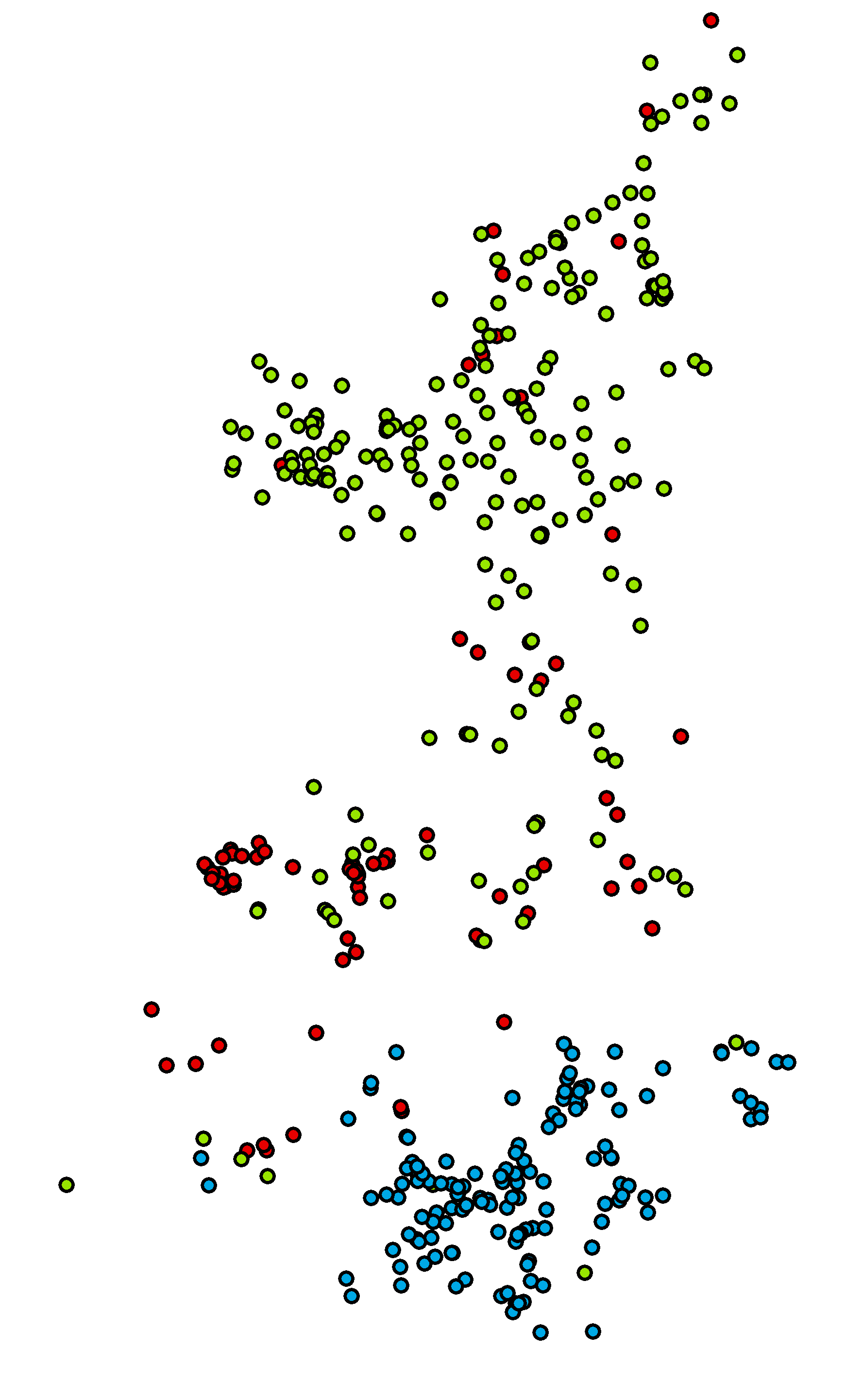

Supplement: S1 File — (ZIP) [file pone.0264238.s001.zip › S1 File. Analysis of the spatial distribution of fort-type settlements in Shaanxi/Cluster distribution map.tif]

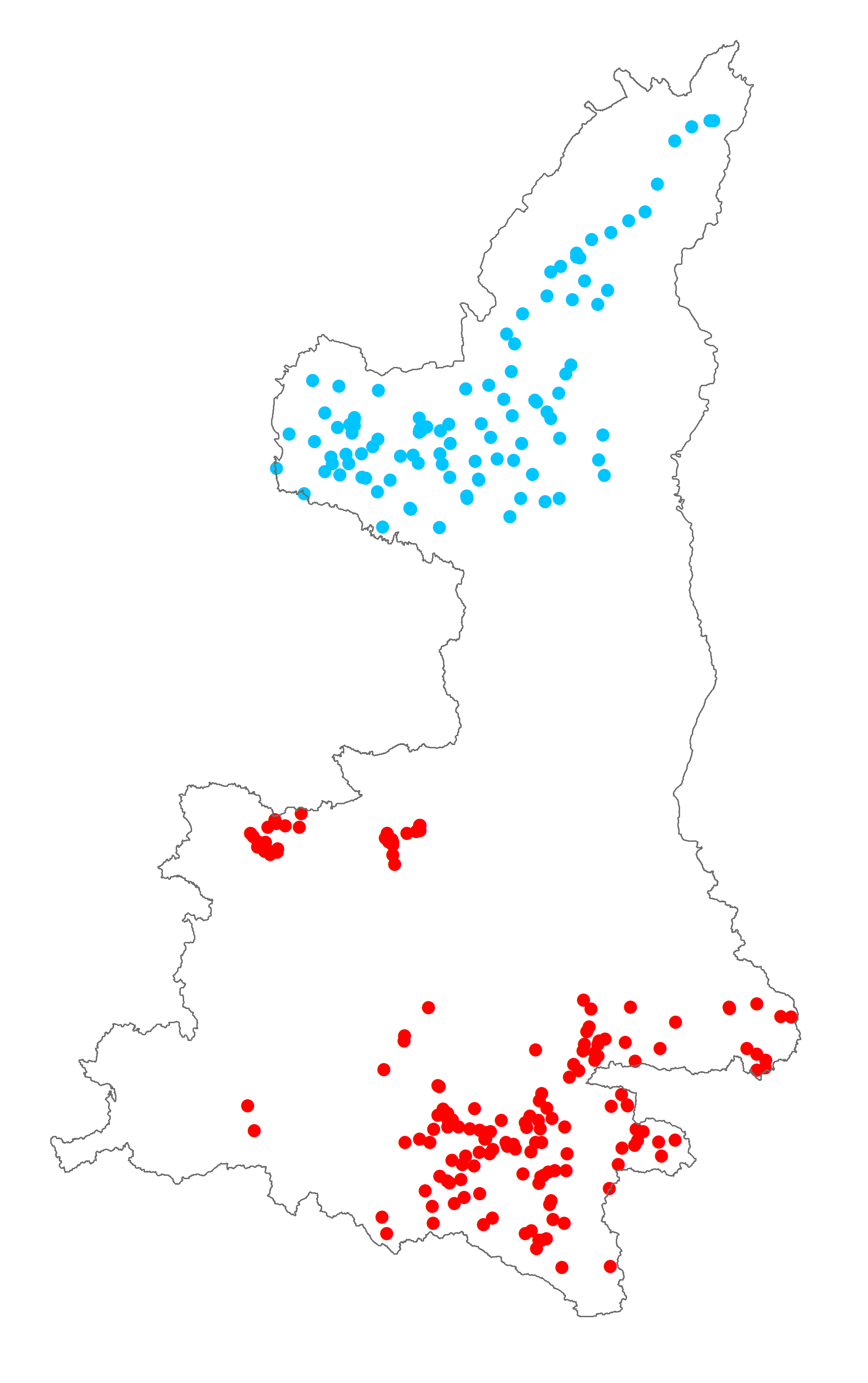

Supplement: S1 File — (ZIP) [file pone.0264238.s001.zip › S1 File. Analysis of the spatial distribution of fort-type settlements in Shaanxi/Distribution map of different construction force.tif]

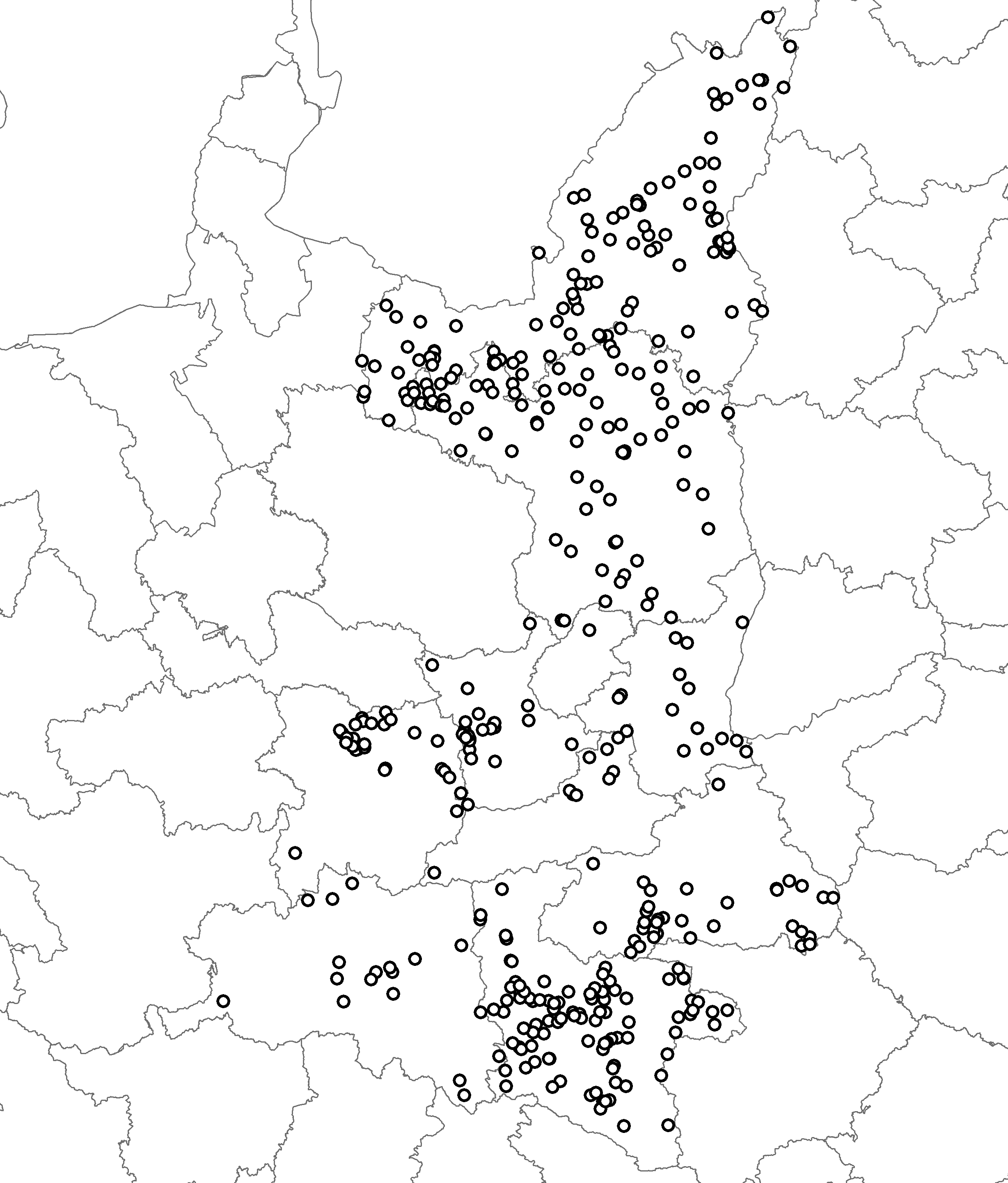

Supplement: S1 File — (ZIP) [file pone.0264238.s001.zip › S1 File. Analysis of the spatial distribution of fort-type settlements in Shaanxi/Distribution map of fort-type settlements in Shaanxi.tif]

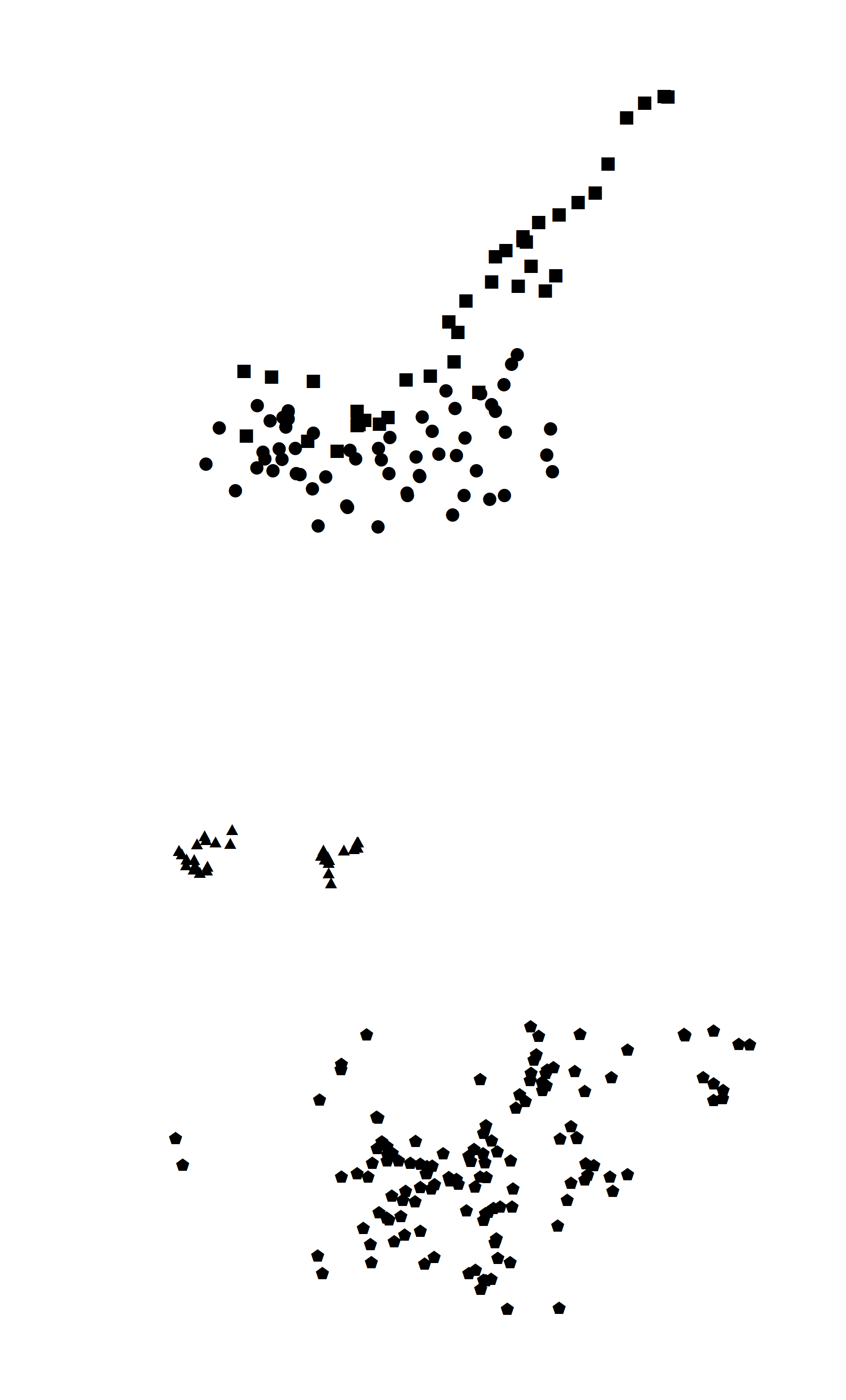

Supplement: S1 File — (ZIP) [file pone.0264238.s001.zip › S1 File. Analysis of the spatial distribution of fort-type settlements in Shaanxi/Distribution map of the four group systems in Shaanxi.tif]

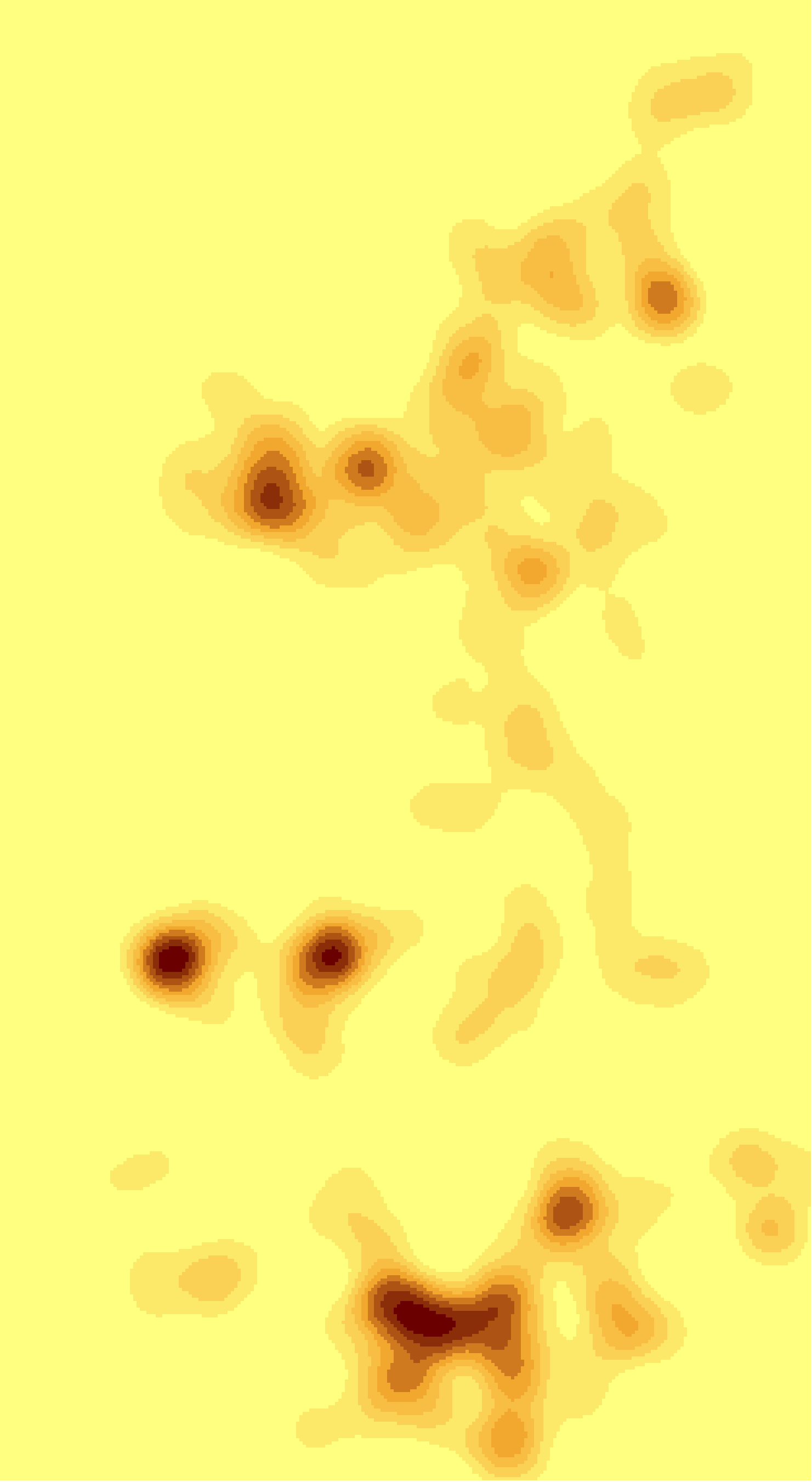

Supplement: S1 File — (ZIP) [file pone.0264238.s001.zip › S1 File. Analysis of the spatial distribution of fort-type settlements in Shaanxi/Kernel density estimation map.tif]

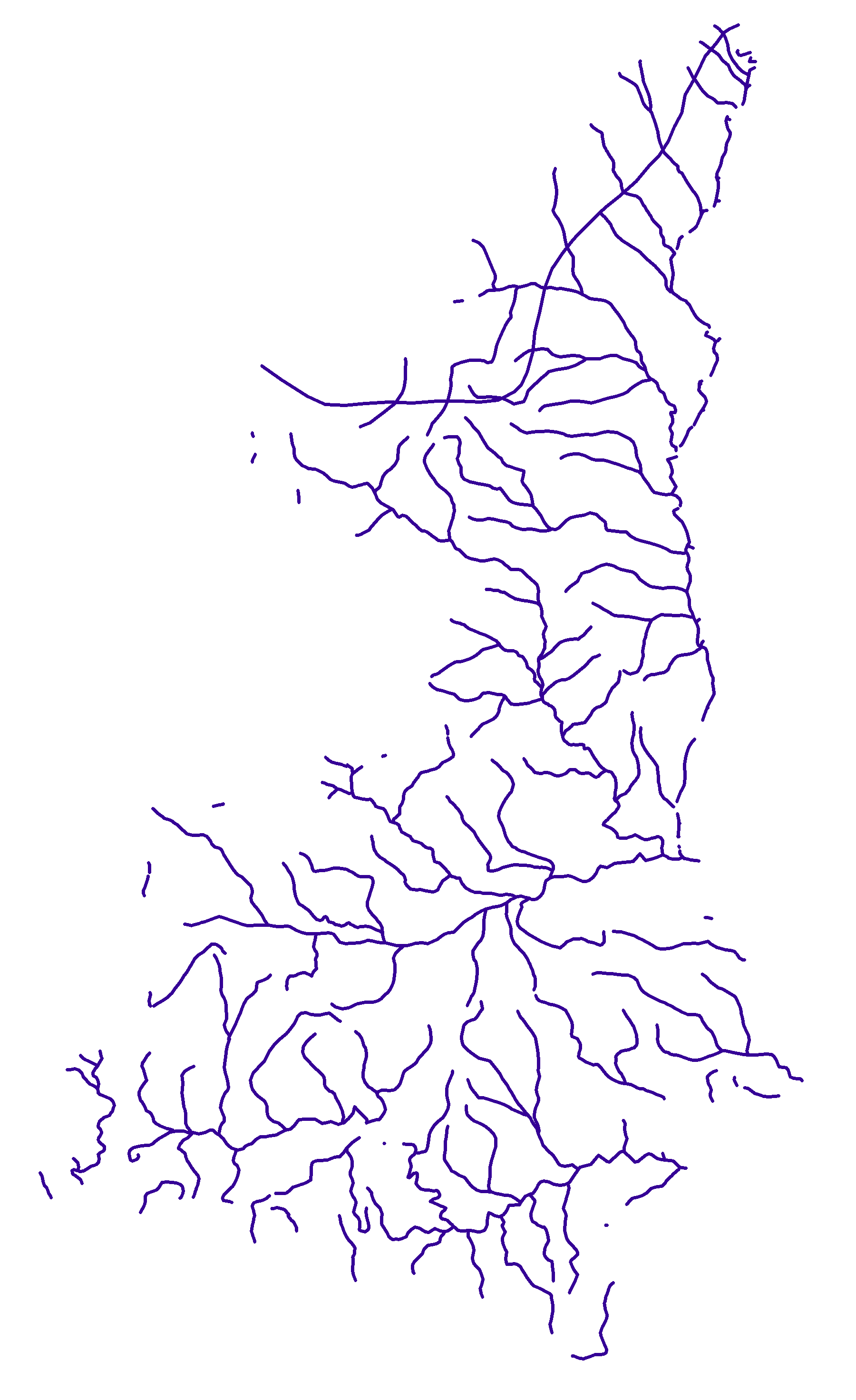

Supplement: S1 File — (ZIP) [file pone.0264238.s001.zip › S1 File. Analysis of the spatial distribution of fort-type settlements in Shaanxi/Line of river and the Great Wall.tif]

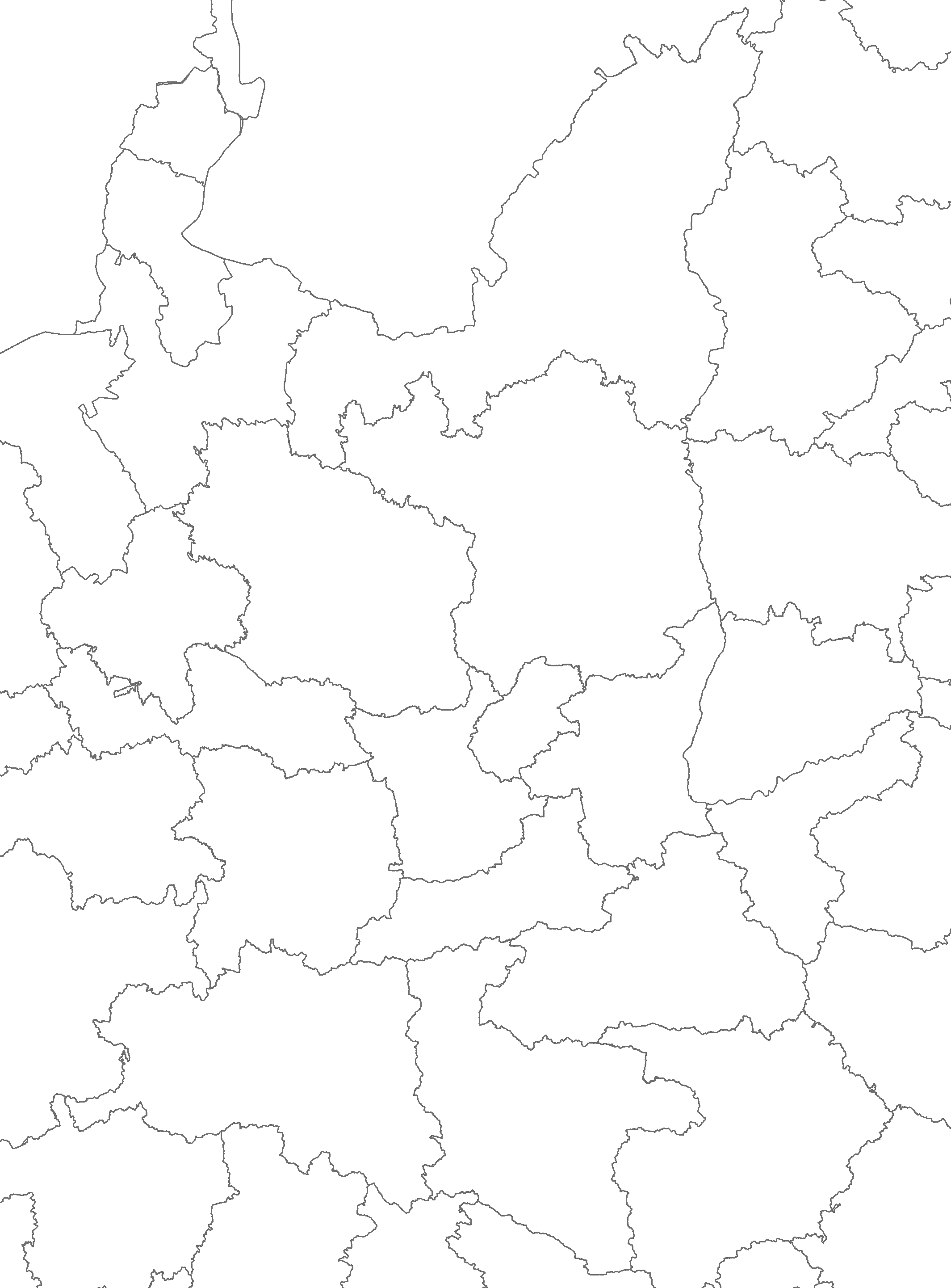

Supplement: S1 File — (ZIP) [file pone.0264238.s001.zip › S1 File. Analysis of the spatial distribution of fort-type settlements in Shaanxi/Municipal boundary of Shaanxi.tif]

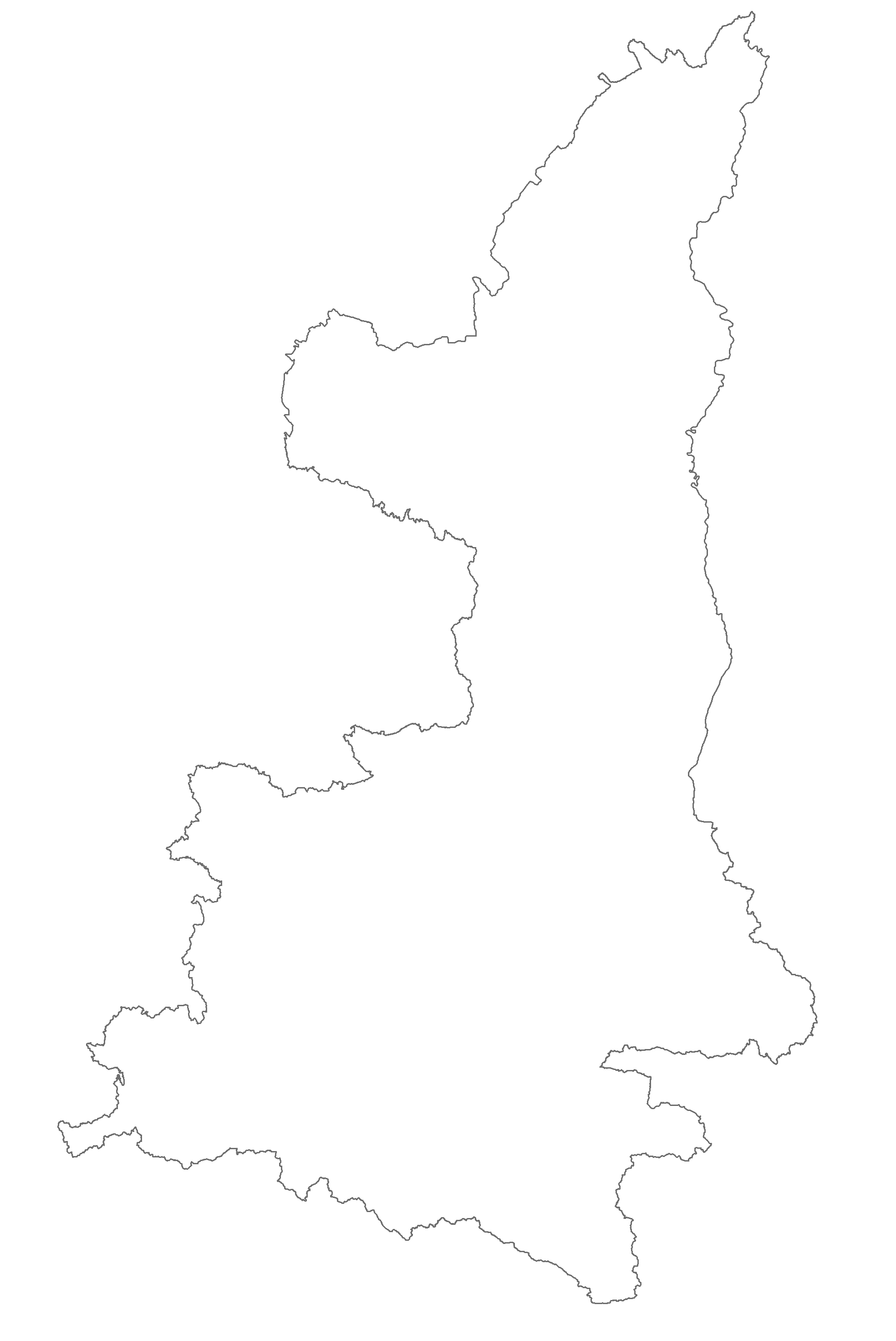

Supplement: S1 File — (ZIP) [file pone.0264238.s001.zip › S1 File. Analysis of the spatial distribution of fort-type settlements in Shaanxi/Provincial boundaries of Shaanxi.tif]

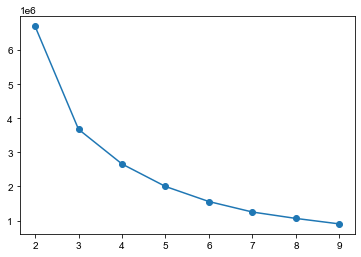

Supplement: S2 File — (TIF) [file pone.0264238.s002.tif]

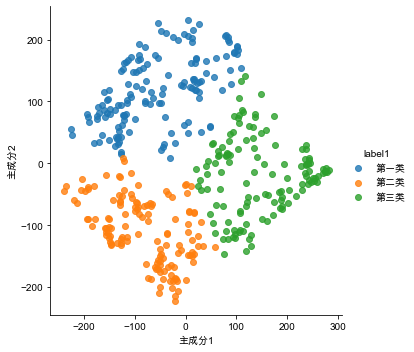

Supplement: S3 File — (TIF) [file pone.0264238.s003.tif]

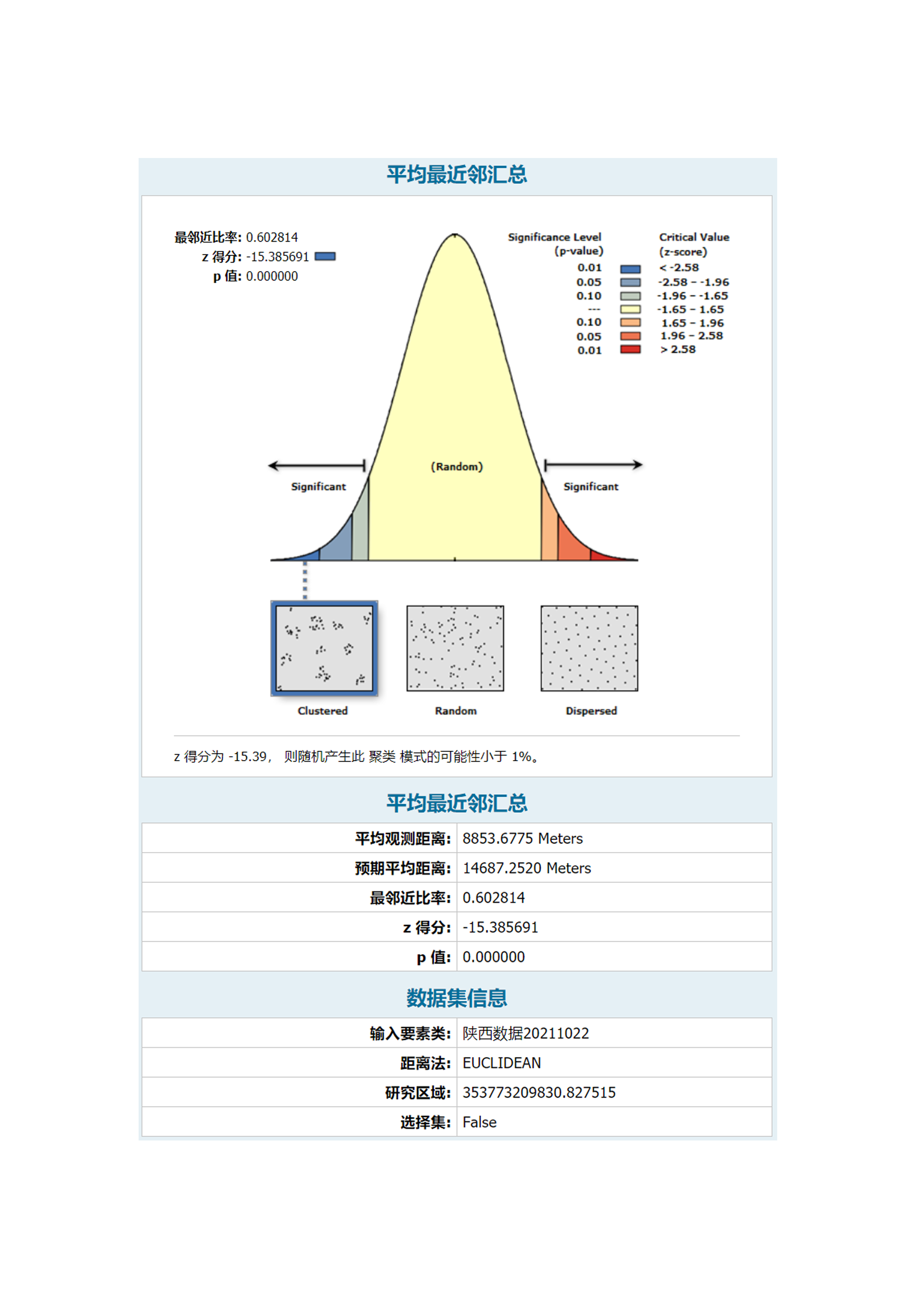

Supplement: S4 File — (ZIP) [file pone.0264238.s004.zip › S4 File. Nearest Neighbor Index Analysis Chart/data of fort-type settlements in Shaanxi.tif]

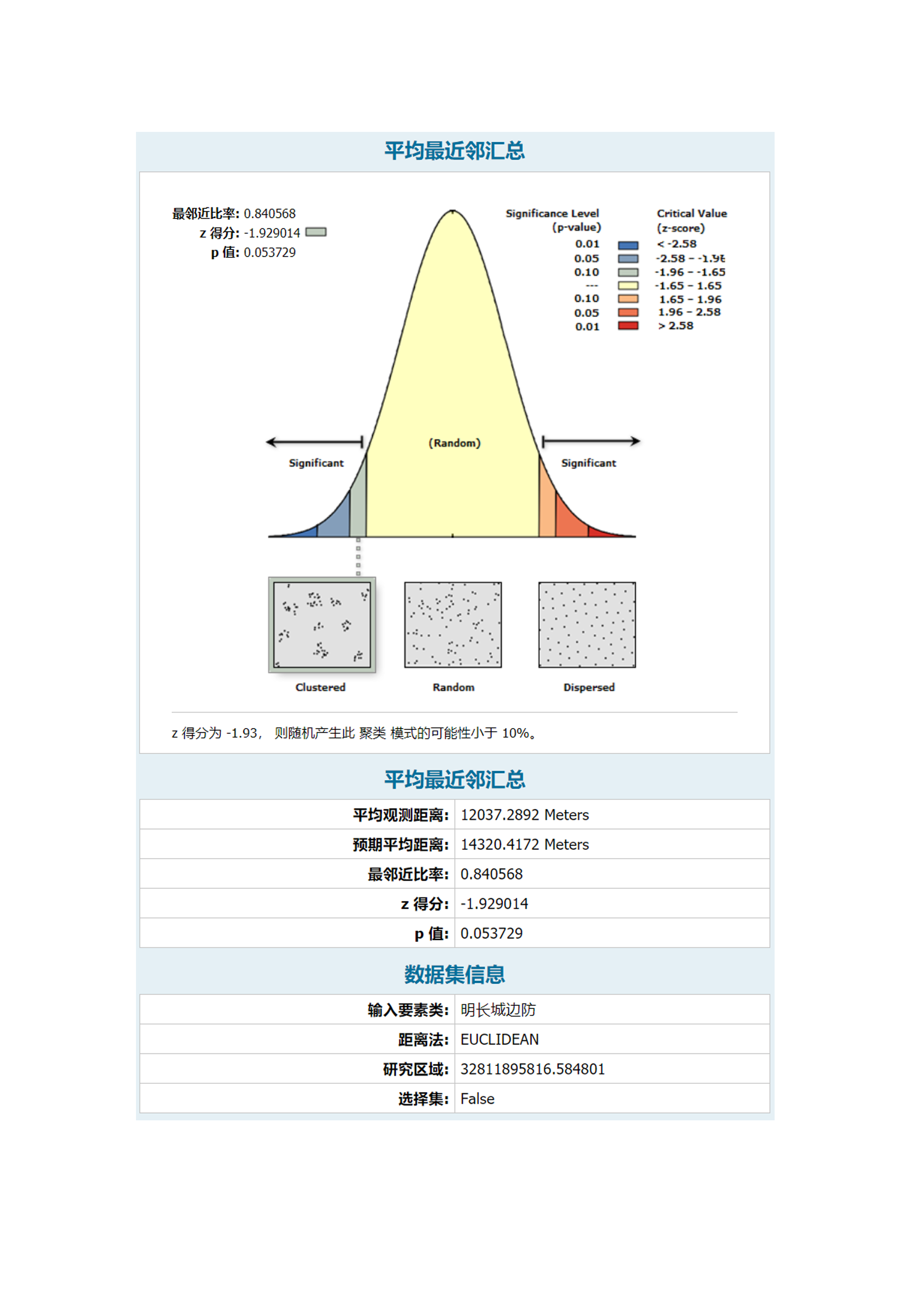

Supplement: S4 File — (ZIP) [file pone.0264238.s004.zip › S4 File. Nearest Neighbor Index Analysis Chart/data of Ming Great Wall military defense system fort cluster.tif]

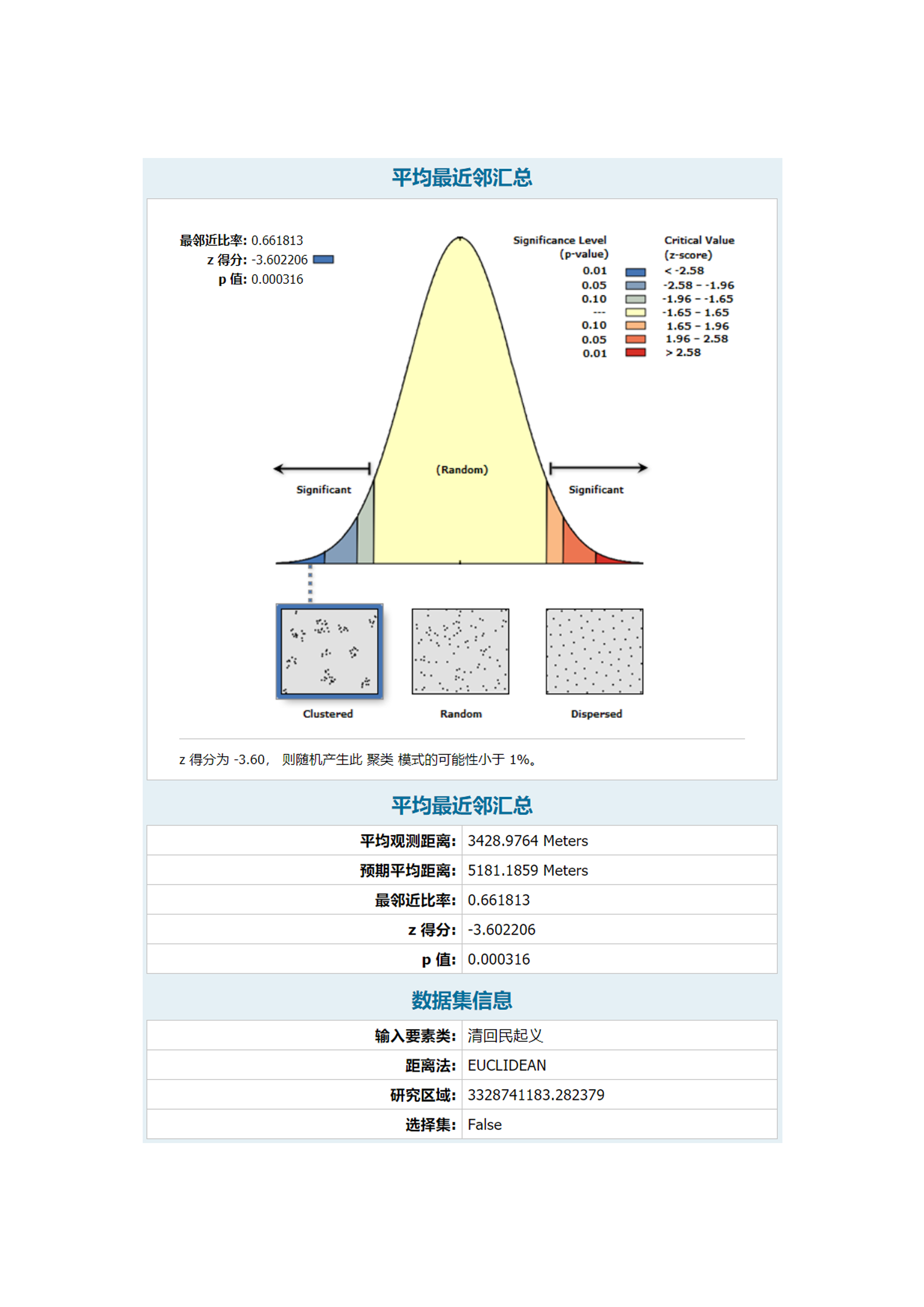

Supplement: S4 File — (ZIP) [file pone.0264238.s004.zip › S4 File. Nearest Neighbor Index Analysis Chart/data of Qing Muslim Uprising democratic earth fort cluster.tif]

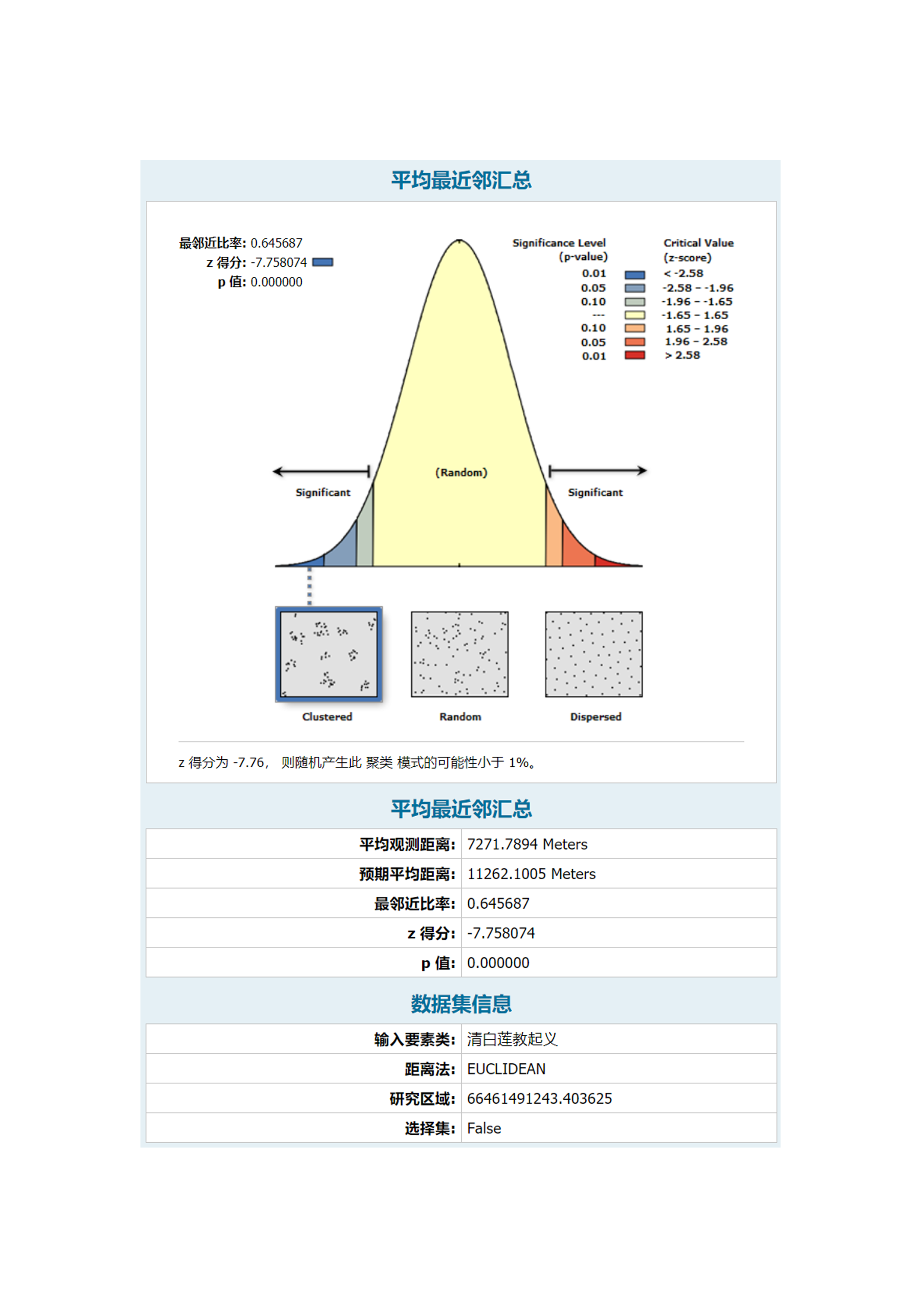

Supplement: S4 File — (ZIP) [file pone.0264238.s004.zip › S4 File. Nearest Neighbor Index Analysis Chart/data of Qing White Lotus Uprising democratic fort cluster.tif]

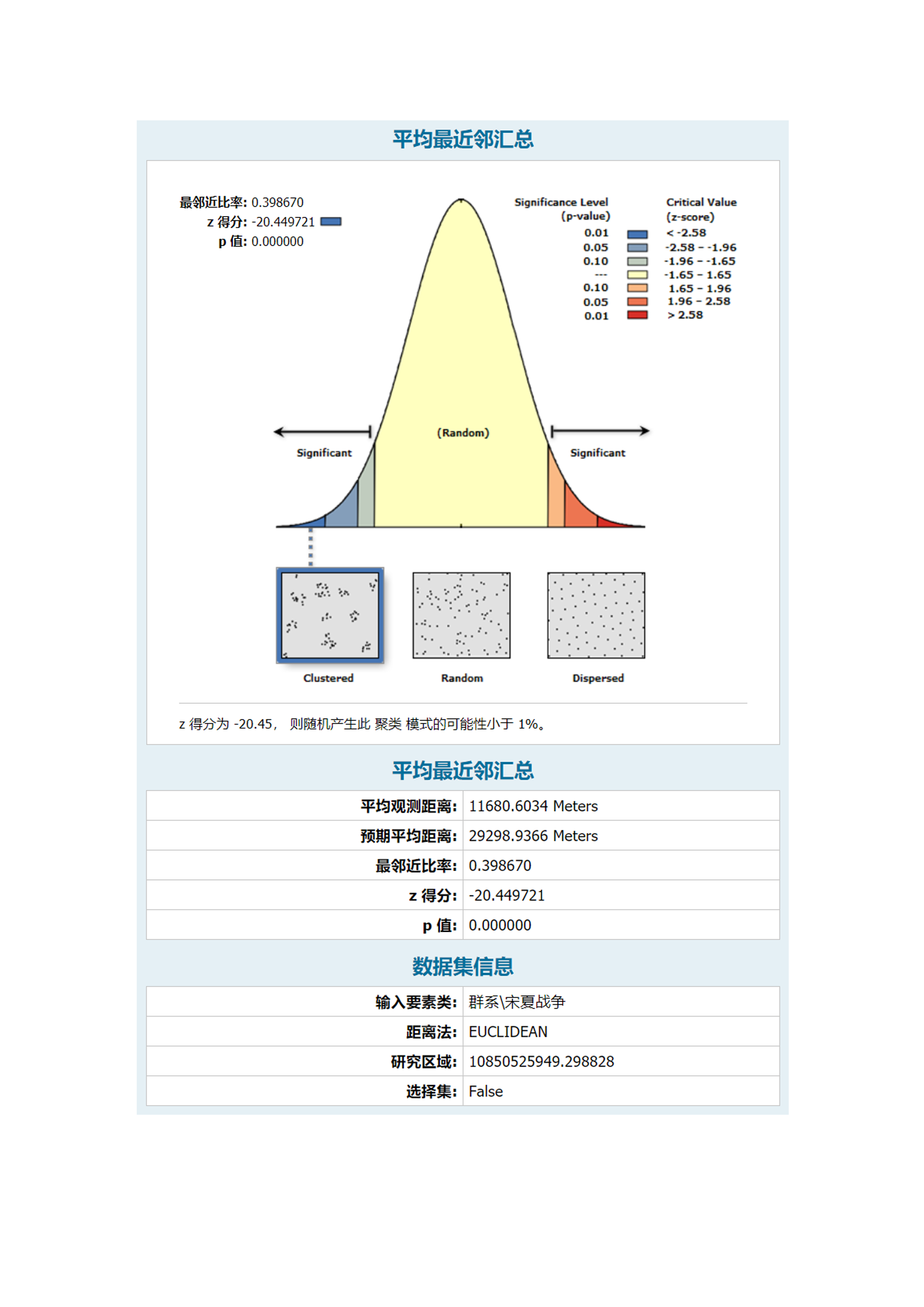

Supplement: S4 File — (ZIP) [file pone.0264238.s004.zip › S4 File. Nearest Neighbor Index Analysis Chart/data of Song-Xia War border military fort cluster.tif]

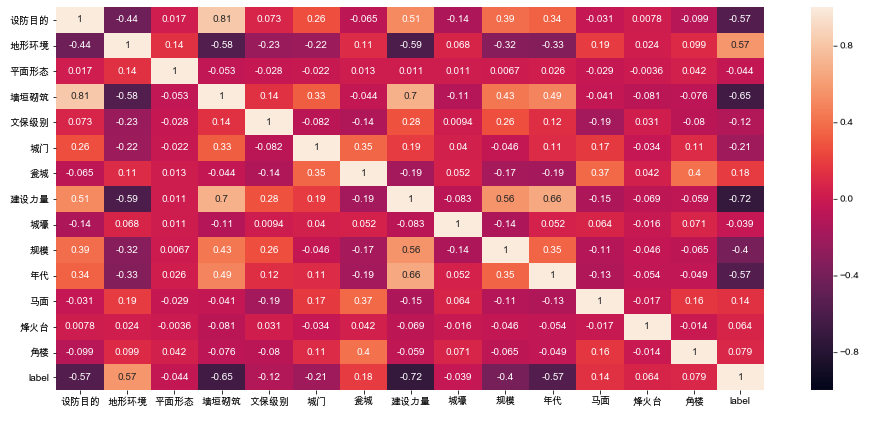

Supplement: S5 File — (TIF) [file pone.0264238.s005.tif]

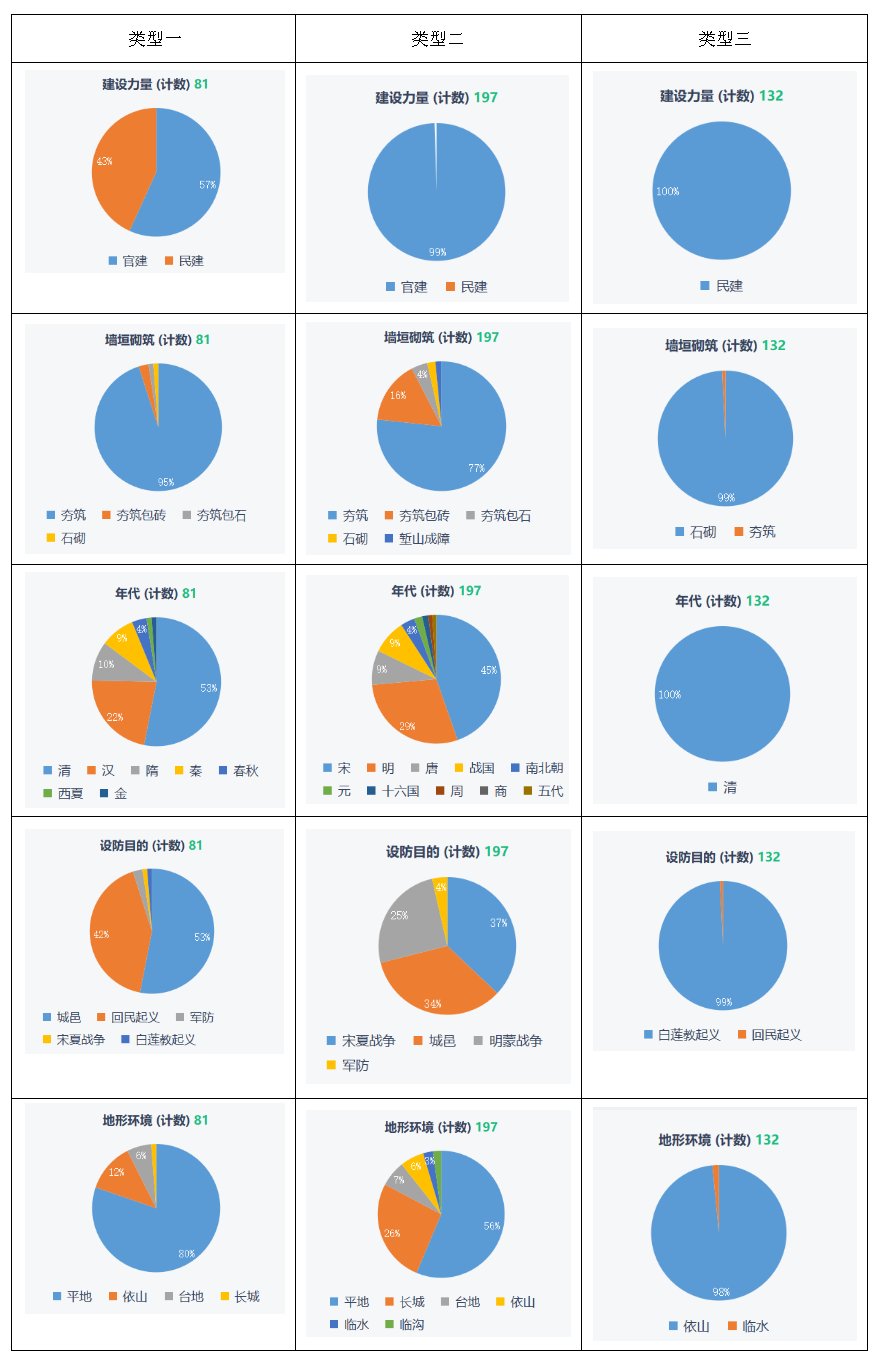

Supplement: S6 File — (TIF) [file pone.0264238.s006.tif]

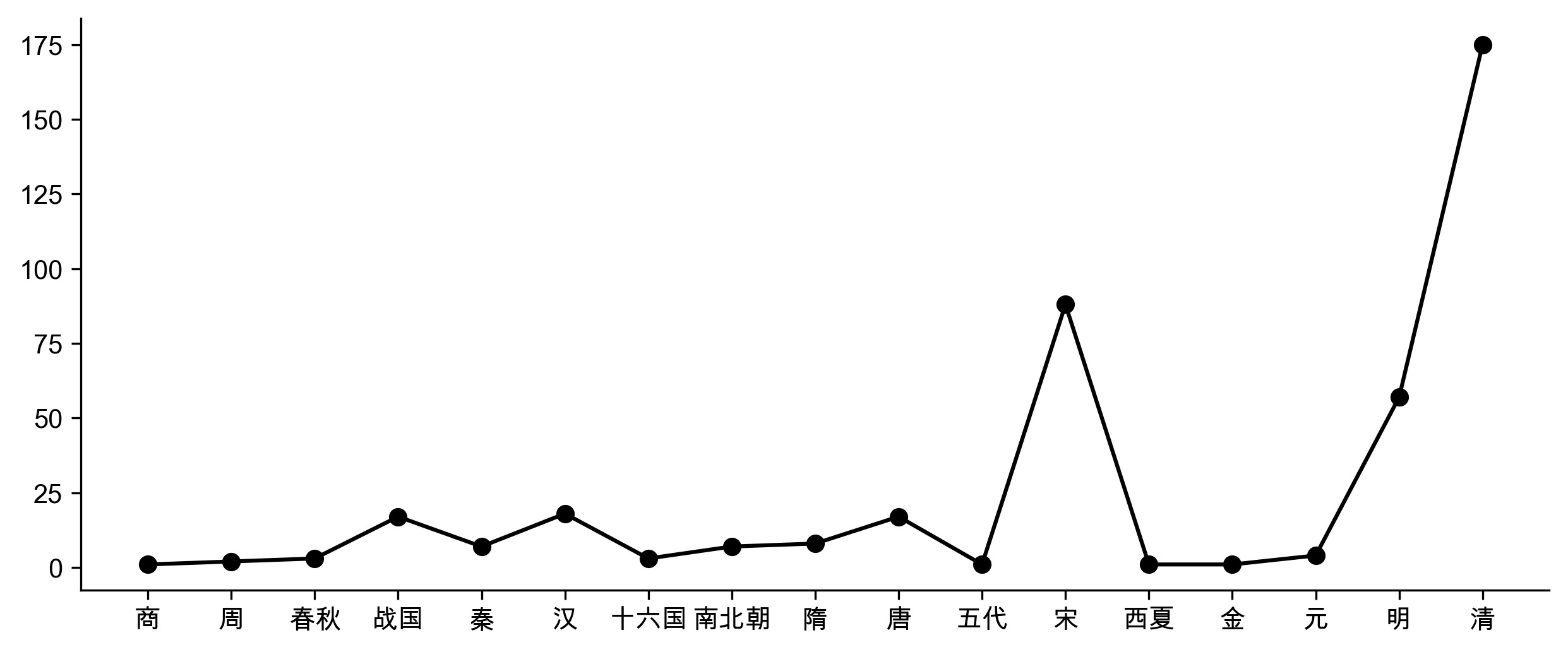

Supplement: S7 File — (TIF) [file pone.0264238.s007.tif]
